# Dynamics of inequality in child under-nutrition in Ethiopia

**DOI:** 10.1186/s12939-021-01478-3

**Published:** 2021-08-14

**Authors:** Mengesha Yayo Negasi

**Affiliations:** Department of Business and Management, Addis Ababa Science and Technology University, P.O.Box: 16417, Addis Ababa, Ethiopia

**Keywords:** Child, Undernutrition, Dynamics of inequalities, Ethiopia, F22, I15, O15

## Abstract

**Introduction:**

Although Ethiopia has already achieved a remarkable progress in reducing under-five mortality in the last decades, undernutrition among children is still a common problem in this country. Socioeconomic inequalities in health outcomes in Ethiopia have been thus of focus in academia and policy spheres for a while now. This study provides new evidence on child undernutrition inequalities in Ethiopia using longitudinal perspective.

**Method:**

Using three round of household panel survey (from 2012 to 2016), we use concentration index (associated curve), different mobility index approaches for measuring inequalities and its dynamics, and decomposition method to identify contributing factors.

**Results:**

In all concentration index computing approaches and socioeconomic status ranking variables, the concentration indices are significant with negative value. This implies that in either of short-run or long-run inequality estimates, the burden of unequal distribution of undernutrition remains on the poor with significant difference across regions. While employing different SES ranking variables, the difference in the concentration indices is only found significant in case of Height-for-age Z-score. It signifies that relatively higher inequality is measured using consumption as ranking variable. Significant difference in inequality is also shown across regions. With respect to dynamics of inequalities, results on mobility indices computed based on Allanson et al. (Longitudinal analysis of income-related health inequality. Dundee Discussion Working Paper No. 214, 2010) approach show that inequality remain stable (persistent) in Height-for- age Z-score, and reduction of inequality in Weight-for- age Z-score while in case of Weight-for- height Z-score, there is no clear trend over subsequent waves. Results on decomposition of inequalities show that the major contributors are wealth index, consumption and mother’s education.

**Conclusion:**

The argument of the choice of welfare indicator can have a large and significant impact on measured socioeconomic inequalities in a health variable which it depends on the variable examined. Employing longitudinal perspective rather than weighted average of cross-sectional data is justifiable to see the dynamic of inequality in child malnutrition. In both socioeconomic status ranking variables, the bulk of inequality in malnutrition is caused by inequality in socioeconomic status in which it disfavours the poor in both cases. This calls for enhancing the policy measures that narrow socioeconomic gaps between groups in the population and targeting on early childhood intervention and nutrition sensitive.

## Introduction

Child malnutrition continues to be the leading public health problem in developing countries. Globally, there were 165 million stunted, 99 million underweight, and 51 million wasting children by year 2012. It kills 3.1 million under-five children every year [1]. Undernutrition among children is a critical problem because its effects are long lasting and go beyond childhood. It has both short and long- term consequences [2, 3]. Ethiopia has the second highest rate of malnutrition in sub-Saharan Africa (SSA). The country faces the four major forms of malnutrition: acute and chronic malnutrition, iron deficiency anaemia, vitamin A deficiency, and iodine deficiency disorder [4].

Although Ethiopia has already achieved a remarkable progress in reducing under-five mortality in the last decades, undernutrition among children is still a common problem in this country. Undernutrition can best be described in the country as a long- term year round phenomenon due to chronic inadequacies in food combined with high levels of illness in under-five children. It is the underlying cause of 57% of child deaths [5]. Thus, socioeconomic inequalities in health outcomes have been of focus in academia and policy spheres for a while now. The vast empirical literature in the area, however, is mixed and context-specific. Many recent papers pursue a cross-country path, documenting widening inequalities in some countries and improvements in others. For example, Wagsta et al. [6], based on Demographic Household Survey (DHS) data from 64 developing countries, find that the poor are more likely to face health risks, including child undernutrition and mortality, and less likely to receive key health services. They conclude that health outcomes are pro-rich while health interventions such as vaccinations are pro-poor.

Studies from low income countries reveal similar mixed conclusions (for example, [7–9]). After reviewing vast literature and data from nearly 100 low and middle income countries, Barros et al. [7] find that poor children and their mothers lag well behind the better-off in terms of mortality and under nutrition. In contrast, they note that poor children are less obese and more adequately breastfed than their rich counterparts. McKinnon et al. [8] also analyze wealth-related and educational inequalities in neonatal mortality (NMR) for 24 low- and middle-income countries and find substantial heterogeneity in both magnitude and direction of NMR inequalities between countries. They note that while inequalities declined in most of the countries, pro-rich inequalities increased in a few countries, including Ethiopia. Quentin et al. [9] compare inequalities in child mortality and their trends across 10 major African cities including the Ethiopian capital, Addis Ababa. Using DHS data by computing both absolute (difference and Erreyger’s index) and relative inequality (rate ratio and concentration index) measures, they reveal significant inequalities in four of the 10 cities including Addis Ababa in the most recent survey.

The multi-country studies highlighted earlier and many others can provide useful insight into inequalities in child health outcomes. However, for an in-depth scrutiny of the issue, a country-level study would offer more as it takes into account the specific contexts of the country under investigation. To this end, there are various reasons why Ethiopia could be an interesting case study on inequalities in child health outcomes. Firstly, the government of Ethiopia over the past decade and half has enacted various strategies and plans in the health sector to expand health infrastructure [10]. Nonetheless, the country has not yet met all the international benchmarks established by the WHO for various indicators in addition to issues related quality of health services. Secondly, Ethiopia has been a focus of many in relation to its commitments to achieve child health-related Millennium Development Goals (MDGs). Although Ethiopia has already achieved a remarkable progress in reducing under-five mortality in the last decades, undernutrition among children is still a common problem in this country. To reverse the situation, it still requires that further efforts using a more policy-relevant measure of inequality taking a longitudinal perspective (dynamics aspect). Lastly, there are various household- and child-level surveys in Ethiopia. In addition to the traditional DHS, there are Young Lives Survey and the Ethiopia Socioeconomic Survey (ESS). Launched by the World Bank and the country’s CSA in 2011, the ESS contains selected child health outcome indicators and is superior to the DHS in terms of containing consumption expenditure and providing panel data (of three rounds in 2011/12, 2013/14 and 2015/16). Given those facts, conducting study on inequality of health outcome using different welfare indicators and longitudinal aspect is relevant to get updated evidences for formulating appropriate and timely policy.

In fact, there are few previous studies that explore child health outcome inequalities in Ethiopia such as [11–17]. Estimates from a World Bank [18] fact sheet on health equity and financial protection on the country show progress over the 2000–2011 periods on a host of child health indicators such as stunting, underweight, diarrhea, fever, etc. However, these DHS-based estimates reveal increased pro-poor inequalities over time. A recent study that is of high relevance to our case is Ambel et al. [12]. They analyze child (and maternal) health inequalities using DHS data from 2000 to 2014. Very recently, Alemu et al. [11] provide a spatial analysis of all standard indicators of undernutrition and identify hotspot locations in the country. Hailie et al. [15] do the same but only for stunting and identify the determinants of inequality using multi-level regression.

Most of the aforementioned empirical evidences on inequalities in child health outcomes are using cross -sectional such as DHS data and various national surveys. However, previous DHS-based studies have been constrained by the lack of expenditure data. In a predominantly rural society such as Ethiopia, measuring household economic status by a stock variable i.e. wealth index is questionable while analyzing such issues as inequalities in child undernutrition. It is fact that aggregate consumption may well be a better indicator of household welfare than the DHS wealth index because it may not respond quickly to shocks. Again, this implies that the choice of welfare indicator can have a large and significant impact on measured socioeconomic inequalities in a health variable. Moreover, the growing number of countries with longitudinal^1^ data sets comprising socioeconomic and health related information has stimulated the development and refinement of different approaches to the measurement of health inequalities. It indicates that we need more sophisticated approaches to monitor inequalities and design appropriate policy interventions because longitudinal measures are required to determine the incidence and effectiveness of interventions designed to tackle such health inequalities in the population^2^. Nonetheless, analyzing inequalities in child health outcome using alternative welfare indicators such as consumption and panel estimation^3^ is not common or limited in many studies, especially in Ethiopia.

In this paper, we provide a more policy-relevant measure of inequality taking a longitudinal perspective to analyze dynamics of child undernutrition inequalities in Ethiopia, focusing only on children under five ages. This study differs from the previous literature (with specific to Ethiopia’s case) in that it uses a flow measure consumption expenditure (data with good-quality nationally-representative household consumption surveys from the World Bank’s Living Standards Measurement Study, LSMS), missing in DHS to investigate inequalities in child undernutrition while still supplementing it with wealth index. It also examines spatial aspect of inequalities in child malnutrition such as across regions and rural-urban. Besides, unlike previous DHS-based studies, the current study employs panel data trend analysis on the inequalities from similar children tracked by the three rounds of the ESS from 2011 to 2016. Moreover, to address the short-run and long-run situation of inequality, analysis on dynamics of inequalities in child malnutrition over time using different approaches for mobility indices is considered. The key results of this study show that inequality in undernutrition varies while we use different socioeconomic status (SES) indicators (such as wealth index and consumption), i.e. relatively higher inequality is observed in case of consumption as SES ranking variable. Results on inequality using spatial aspect signify that significant difference in inequality of undernutrition is shown across regions. In terms of dynamics inequality, persistence of inequality in undernutrition-stunting is seen. Our inequality results are robust to different measurement scale, inequality aversion parameters/distributional sensitivity parameters, symmetric concentration index or ‘sensitivity to extremity. Those results are also standardized for age and sex.

The rest of the study is organized as follows; in section two, comprehensive literature review on inequality in child health outcome is presented. Section three covers a brief discussion of methods, data sources and variables measurement. Section four provides results and analyses on inequalities in child malnutrition, dynamics of socioeconomic related inequality using mobility indices, decomposition of inequality to major contributing factors and different robustness of results. Last section puts some concluding remarks and policy implication.

## Literature review

To have better understanding on the dynamic relationship or interaction between socioeconomic and other factors, and health outcomes, it is noteworthy to adopt multidimensional conceptual framework. One of such a framework is developed by Wagsta [20] in which it states that health outcomes are subject to different factors such as household and communities, health service and systems, supply side factors and policies which have multidimensional or dynamic nature. There are also alternative frameworks that can be used to describe the complex range of factors that influence child nutrition. One that is widely cited is the United Nations Children’s Fund (UNICEF) framework for improving child nutrition, which was developed a couple of years ago. As of Thomson et al. [21], at the core of this framework, there are a number of direct determinants of nutrition, called `immediate’ causes, followed by a further group called `underlying’ causes and, at the periphery, a group of `basic’ causes. Basic causes include political, ideological, economic, environmental, resource and technology factors. The UNICEF framework describes `short-route’ interventions that address the immediate causes and `long-route’ interventions that address underlying and basic causes.

There are dozens of empirical findings applied to assess health outcome, particularly the inequality of child health outcome. Basically, they vary in methods/approaches, and data type. Some use cross-sectional while others though limited and at macro level, apply panel data approach. They also differ in following either bivariate-descriptive approach or multivariate-causal analysis. However, some very relevant works are covered here.

One of the debating on health outcome inequalities is on the approach applied to measure inequality. In this regard, Wagsta et al. [22] offer a critical appraisal of the various methods employed to date to measure inequalities in health. However, they suggest that that only two of these--the slope index of inequality and the concentration index are likely to present an accurate picture of socioeconomic inequalities in health. Kakwani et al. [23] also contribute on inequality measurement by looking at standardizing using demographic factors (like age and sex) play a vital role on socioeconomic inequality analysis in health.

Jones and Lobez [24] presents a method for the measurement of changes in health inequality and income-related health inequality over time in a population. However, Allanson et al. [25] elucidate the nature of the Jones and Lopez [24] index of “health-related income mobility” and explains the negative values of the index that have been reported in all the empirical applications to date. They further question the value of their index to health policy makers and proposes an alternative index of “income-related health mobility” that measures whether the pattern of health changes is biased in favour of those with initially high or low incomes. They illustrate their work by investigating mobility in the General Health Questionnaire measure of psychological well-being over the first nine waves of the British Household Panel Survey from 1991 to 1999.

Specifically, with regard to malnutrition inequalities, although many surveys of children have been conducted since the 1970s, lack of comparability between them has made it difficult to monitor trends in child malnutrition. To this end, DeOnis [26] demonstrates that analysis of cross- sectional data from 241 nationally representative surveys in a standard way to produce comparable results of low height-for-age (stunting). He then documents that despite an overall decrease of stunting in developing countries, child malnutrition still remains a major public health problem in these countries. In some countries, rates of stunting are rising, while in many others they remain disturbingly high. Moreover, using decomposition method, Wagsta et al. [27] show that inequalities in height-for-age in Vietnam in 1993 and 1998 are largely accounted for by inequalities in consumption and in unobserved commune-level influences. They add that rising inequalities are largely accounted for by increases in average consumption and its protective effect, and rising inequality and general improvements at the commune level. Although it seems superior in using consumption rather than wealth index for ranking household position based on their socioeconomic status, this study is still subject to the usual caveats regarding the causal interpretation of cross-sectional results and also unable to see the long-run inequality situation. Using cross- sectional data sets available from the DHS of 15 countries in SSA, Fotso [28] also notes that though socioeconomic inequalities in stunting do exist in both urban and rural areas across countries in SSA, they are significantly larger in urban areas.

Many recent papers also follow a cross-country path, documenting widening inequalities in some countries and improvements in others (for instance, [6–8], and [29]). For example, using original data from 131 DHSs and 48 multiple indicator cluster surveys from 1990 to 2011, Bredenkamp and Ellen [29] examine trends in socioeconomic inequalities in stunting and underweight, as well as the relationship between changes in prevalence and changes in inequality. Then, they infer that reductions in the prevalence of undernutrition have generally been accompanied by neither widening nor narrowing inequalities. It rather indicates that the picture is one of a strong persistence of existing inequalities. Barros et al. [7] and McKinnon et al. [8] also demonstrate similar results. However, to see such kind of dynamics of inequality, panel data is more appropriate than one time snapshot data. Other empirical works from developing countries show similar conclusions.

For an in-depth scrutiny of the issue, a country-level study would offer more as it takes considers the specific contexts of the country under investigation. To this end, only few previous studies explore child health outcome inequalities in Ethiopia. Using Ethiopian DHS cross-sectional data from the 2000, 2005 and 2011, Skaftun et al. [30] compute concentration index and a geographic Gini index to measure inequality. Then, they report that significant pro-rich inequalities were found for all indicators except treatment for suspected pneumonia in 2011. The socioeconomic inequalities seem to increase from 2000 to 2011 for under-five and neonatal deaths, whereas they are stable or decreasing for the other indicators. More importantly, Ambel et al. [12] analyze trends in child (and maternal) health inequalities by household wealth status, mothers’ education, and place of residence in Ethiopia. Using cross-sectional DHS data from 2000 to 2014, they compute concentration indices (CIs) in three undernutrition indicators (stunting, wasting and underweight) and show that widening pro-rich inequality. Trend-wise, they report that inequalities more than doubled for all undernutrition indicators over the survey periods. These findings show the issue of inequality in child health outcomes should be a concern of research and policy in Ethiopia.

In summary, as it is aforementioned at the outset of the empirical literature section, the existing literature on the area under this study differs in many ways, even those findings are mixed. They are subject to number of critics. Previous DHS-based studies have been constrained by the lack of expenditure data. In a predominantly rural society such as developing countries, particularly Ethiopia, measuring household economic status by a stock variable i.e. wealth index is questionable^4^ while analyzing such issues as inequalities in child undernutrition. This is due to the fact that the choice of welfare indicator might have a large and significant impact on measured socioeconomic inequalities in a health variable which it depends on the variable examined. In terms of data type also, all employ a cross-sectional data for specific context. However, for those who are interest looking at long-run inequality compare to short-run one and policy formulation, rely on cross-sectional evidence is not warranted. It is true that the determination of health is essentially a dynamic process; health today reflects experiences of the past. Hence, applying longitudinal data is superior.

Thus, to the best of my knowledge, this study is different from the previous literature in particular to Ethiopia, in that it uses flow measure consumption expenditure, missing in DHS to investigate trend and magnitude of inequalities in child undernutrition while still supplementing it with wealth index. Moreover, unlike previous studies which use DHS and other data sets, the current study provides a panel data trend analysis on the inequalities from similar children tracked by the three rounds of the ESS from 2011 to 2016. Then, for dynamics of inequalities in child undernutrition, we employ different mobility index computing approaches, and thereby see whether the cross-sectional (short-run) evidences on inequality overestimate or underestimate the long-run inequality picture.

## Method and data

### Data

Data for the study comes from the ESS collected jointly by the CSA of Ethiopia and the World Bank as part of the Living Standard Measurement Study-Integrated Surveys on Agriculture (LSMS-ISA). It is a longitudinal survey with three waves (2011/12, 2013/14 and 2015/16). The ESS^5^ sample is a two-stage probability sample. It employs a stratified, two-stage design where the regions of Ethiopia serve as the strata. The first stage of sampling entails selecting enumeration areas (i.e. the primary sampling units) using simple random sampling (SRS) from the sample of the Agriculture Sample Survey (AgSS) enumeration areas (EAs). The AgSS EAs were selected based on probability proportional to size of population (PPS). The sample design of the first wave provides representative estimates at the national level for rural-area and small-town households while subsequent waves include large towns and cities. The samples are also regionally representative for the major regions of the country (Oromia, Amhara, Tigray, and SNNP) as well as Addis Ababa since the second wave. The second stage of sampling is the selection of households to be interviewed in each EA.

The surveys provide household-level data on a range of issues such as consumption expenditure, assets, food security shocks, copying strategies, non-farm enterprises, credit. Very importantly, individual- level data are available on socioeconomic, demographics, education, health and time use (labor and leisure). Moreover, as traditional in LSMS surveys, community-level data on a host of issues such as health infrastructure as well as market price data from two nearest local markets are collected. Finally, data are obtained from 3969, 5262 and 4954 households in the first, second and third waves respectively. However, the sample for health variable data is restricted to children whose age is below 5 years, which is considered in this study.

#### Health outcome variable

Our health outcome interest is malnutrition using anthropometric indicator. Theoretically, the body of a child responds to malnutrition in two ways that can be measured by anthropometric survey. First, a reduction in growth over the long-term results in low height-for-age or stunting. Second, a short-term response to inadequate food intakes is assessed by weight relative to height (wasting). The combination of short-term and long-term food shortage and growth disturbances produces low weight-for-age (underweight) (ONIS, 2000). Survey data often contain measures of weight and height, in particular for children. Weight and height do not indicate malnutrition directly. Besides age and sex, they are affected by many intervening factors other than nutrient intake, in particular genetic variation. However, even in the presence of such natural variation, it is possible to use physical measurements to assess the adequacy of diet and growth, in particular in infants and children. This is done by com-paring indicators with the distribution of the same indicator for a healthy reference group and identifying extreme or abnormal departures from this distribution [31].

Irrespective of what particular reference data are used, anthropometric indices are constructed by com-paring relevant measures with those of comparable individuals (in regard to age and sex) in the reference populations. There are three ways of expressing these comparisons: Z-score (standard deviation score), percent of median and percentile. However, the preferred and most common way of expressing anthropometrics indices is in the form of Z-scores. More specifically, Z-score for an individual i is calculated using eq. 1:

1 $$ \mathbf{Z}-\mathbf{scorei}=\left(\frac{{\boldsymbol{X}}_{\boldsymbol{i}\kern0.5em }-{\boldsymbol{X}}_{\boldsymbol{r}}}{{\boldsymbol{\delta}}_{\boldsymbol{r}}}\right) $$

Where *X*_*i* _ is an observed value for *i*^*th*^ child in a target population; *X*_*r*_ is a median of the reference population; and δ is a standard deviation (SD) of the reference population.

Thus, the health outcome variables used in this study are the three anthropometric indicators (Height-for-age Z-score (HAZ), Weight-for-Height Z-score (WHZ), and Weight-for-Age Z-score (WAZ). First, those anthropometric indicators from age, height/length and weight data following the World Health Organization, WHO [32] child growth standards are computed. It is then stated that stunting, wasting and underweight levels for children aged less than 5 years as shown in Table 1.

**Table 1:** List and description of child undernutrition indicators

Indicator	Description
Stunted	If child’s height-for-age z-score is less − 2 standard deviations (SD) from the international median [32] healthy reference group
Wasted	If child’s weight-for-height z-score is less −2 standard deviations (SD) from the international median [32] healthy reference group
Under-weighted	If child’s weight-for-age z-score is less −2 standard deviations (SD) from the international median [32] healthy reference group

#### Other variables

Those are used as explanatory variables for regression -based decomposition analysis as well as SES ranking variables in computing SES - related health inequalities. Broadly, they can be grouped as child level characteristics, household and community level characteristics. The child level characteristic includes child’s age, age square, sex, and illness. Under household level, wealth index, consumption, mother’s education, toilet facilities^6^ and household sizes are considered. At community level, health facilities, access to safe drink water and spatial dimension such as household’s place of residence in the form of rural urban or regions. Detail on each variable definition and measurement are given in Table 2. However, among those household socioeconomic characteristics, wealth index and consumption are chosen as SES ranking variables for household position in measuring inequalities. Let’s see below in detail how those values are constructed:

**Table 2:** Description and measurement of variables used in decomposition analysis

Variables	Definition/Description	Measurement /type
	Anthropometrics indicators	
HAZ-score	The length/height (in meters) of children 0 months to 59 months of age	Height for age
		Z-score
WHZ-score	The weigh(in kilogram) and height of children 0 months to 59 months of age	Weight for-height
		Z-score
WAZ-score	The weight (in kilogram) children of 0 months to 59 months of age	Weight for age
		Z-score
	Demographic characteristics at individual level	
Age	Age of child	Continuous, in months
Age-square	Child age square	Continuous, in months
Gender	Sex of child	Dummy; 1 if male, 0 otherwise
Child illness incidence	Whether the child has had diarrhea in the last 2 weeks leading up to the interview	Dummy; 1 if yes, 0 otherwise
	Socioeconomic characteristics at household level	
Wealth index	How many of each of the following items does the household own? (housing condition)	Continuous, index computed based on PCA
Consumption	Household’s real annual consumption (food and non food total expenditure) per adult equivalent	Continuous, annual real total per adult equivalent
Mother’s education	What is/was biological mother’s highest educational level completed?	Categorical, Level of certificate completed
Household size	Total number of family members	Continuous
Household size under age 5	Number of under 5 age household members	Continuous
Toilet facility	What type of toilet facilities does the household use?	Categorical
	Community level characteristics	
Health care services	Is there any health post in the surrounding community	Dummy;1 if yes, 0 otherwise
Water availability	Is there water service in the community	Dummy;1 if yes, 0 otherwise
Place of residence	Household residence place (urban-rural, region)	Dummy; 1 if rural 0 if urban

##### Wealth index

Households were asked whether they owned from a list of asset items (such as farm implements, furniture and kitchenware, entertainment and communication equipment, electronic item, personal items) or not^7^. It also considers various indicators of housing condition of household such as walls, roof, and floor of the main dwelling; type of kitchen, cooking and bathing facilities. Then, following the standard approach of assessing economic status of the household, the study uses household asset and housing conditions to compute wealth index using principal component analysis (PCA) while sampling weight is taken in to account. Unlike DHS and other data sets’ wealth index which is constructed from urban-based social and economic amenities and may be measuring more of urban/city condition instead of inclusive socioeconomic status, this study uses ESS data which also includes rural based socioeconomic asset indicators.

##### Consumption^8^

The surveys include questions on expenditure on food and non-food items, food security, shocks, and coping mechanisms. The total consumption expenditure (available from the survey) is constructed from food consumption, non-food consumption and education expenditure. Initially, a common reference period is established for all items, and values are imputed in cases in which they are not available (converted to a uniform reference period for example, a year). Then, it follows three steps in constructing a consumption-based living standards measure: (a) construct an aggregate of different components of consumption, (b) make adjustments for cost of living differences, and (c) make adjustments for household size and composition. Household size and a measure of adult-equivalency 9 are constructed based on scale factors such as categorizing age in to different ranges(13 age categories) for both male and female by allocating different weights for each categories. In addition, it uses a regional price index (for 10 regions), based on the index created by the Ministry of Finance and Economic Development (MoFED) in their Household Consumption Expenditure (HCE) 2010/2011, 2013/14 and 2015/16 reports. Nominal and real per adult equivalent consumption were then calculated, and real consumption was re-scaled to have the same overall mean value as nominal consumption. The calculated per capita amounts winsorised at the 97th percentile for non-zero consumption for each item (for details, see LSMS annual report of each wave, guideline for constructing aggregate consumption). In this study, we also group the households into quintiles based on the wealth index and consumption adjusted by sample weights for nationally representative inferences. Of course, using consumption expenditure as socioeconomic ranking variable has its own drawbacks. One constraint is that households might overestimate their level of consumption expenditure for different reasons. Measurement problem is also another limitation. Here, consumption is considered as flow measurement while wealth index is as stock variable. A flow is a quantity which is measured with reference to a period of time. It has time dimension. However, a stock has no time dimension (length of time) as against a flow which has time dimension. A flow shows change during a period of time whereas a stock indicates the quantity of a variable at a point of time. Thus, wealth is a stock since it can be measured at a point of time, but consumption expenditure is a flow because it can be measured over a period of time. Hence, using consumption expenditure which is a flow variable enable us to exploit the time dimension aspect of the variable. This is again in line with the main intention of the study.

### Method

#### Measures of inequality in child malnutrition

The study aims to examine the child undernutrition inequalities in socioeconomic status and spatial dimensions. For socioeconomic inequalities in child health, we use consumption expenditure and wealth index as alternative welfare measures and see the gap between the worse-off (bottom 60%) and better off (40%) as well as between the poorest (1st quintile) and the richest (5th quintile).

And for the spatial dimension, inequalities are traced between rural and urban children as well as among those in various regions of the country. The study also computes absolute and relative inequalities from rate differences and rate ratios.

When there are only two subgroups to compare, difference and ratio are the most straightforward ways to measure absolute and relative inequality. However, the differences and ratios between different groups do not consider inequalities by the whole population. Hence, concentration curves are used to illustrate the trend of the socioeconomic and spatial inequalities in child undernutrition over time. The concentration curve plots the cumulative proportion of the population ranked by a measure of socioeconomic status (such as an index of household wealth and consumption) against the cumulative proportion of the health measure (undernutrition indicators). If concentration curve lies above the diagonal (45 degree line of equality), it is interpreted as child malnutrition is disproportionately concentrated among the poor and the reverse is true while it lies below line of equality. The study also conducts tests of dominance between concentration curves following the procedures in O'Donnell et al. [34].

Since a concentration curve does not give a measure of the magnitude of inequality that can be com-pared conveniently across many time periods, countries, regions, or whatever groups may be chosen for comparison, the study examines inequalities using CI [23, 34] and with possible extension. The CI is defined as twice the area between the concentration curve and the line of equality (the 45-degree line). It provides a summary measure of socioeconomic related health inequality, i.e. a measure of the extent to which the concentration curve diverges from the diagonal. The convention is that the index takes a negative value when the curve lies above the line of equality, indicating disproportionate concentration of the health variable among the poor, and a positive value when it lies below the line of equality. However, when there is no socioeconomic-related inequality, the concentration index becomes zero.

In this study, with availability of panel data, we follow dynamic approach to measure inequality in health rather than a static one used in cross-sectional data. The basic rationality behind is that longitudinal data are more relevant for policy making analysis. The cross-sectional data, static approach is often used to compare inequality at two different points in time while the panel, dynamic approach is essentially useful when interest lies in the long -run rather short-run inequality (which can be the case for example, policy makers). As Jones and Lopez [24] prove theoretically, looking at a different point in time using short-run CI does not give a complete picture rather in panel, it enables us to follow each individual in every year and have thus a complete picture of their relative evolution.

To this end, there are various ways of expressing the CI algebraically. For the measurement of inequality at one point in time, the study uses the CI stated in eq. 2, that is mostly used in the literature for its convenience. It is derived by ranking the population by a measure of SES and then com-paring the cumulative proportion of health with the cumulative proportion of the population ranked by SES.

2 $$ {\boldsymbol{CI}}_{\boldsymbol{t}}=\frac{2}{\boldsymbol{N}{\overline{\boldsymbol{y}}}_{\boldsymbol{t}}}{\sum \limits}_{\boldsymbol{i}=1}^{\boldsymbol{N}}\left({\boldsymbol{y}}_{\boldsymbol{i}\boldsymbol{t}}-{\overline{\boldsymbol{y}}}_{\boldsymbol{t}}\right)\left({\boldsymbol{R}}_{\boldsymbol{i}}^{\boldsymbol{t}}-\frac{1}{2}\right)=\frac{2}{{\overline{\boldsymbol{y}}}_{\boldsymbol{t}}}\boldsymbol{\operatorname{cov}}\left({\boldsymbol{y}}_{\boldsymbol{i}\boldsymbol{t}},{\boldsymbol{R}}_{\boldsymbol{i}}^{\boldsymbol{t}}\right) $$

Where *y*_*it*_ represents the health level of individual i in period t, and ${R}_i^t$ denotes the relative fractional rank of *i*^*th*^ individual in the distribution of SES in period t; N is the sample size at period t . ${\overline{y}}_t=\frac{\sum \limits_{i=1}^N{y}_{it}}{N}$ is the mean of health of the sample in the period t.

Equation 2 shows that the value of concentration index is equal to the co-variance between individual health (*y*_*i*_) and the individual’s rank $\left({R}_i^t\right)$, scaled by the mean of heath in the population (*y*_*i*_).Then to ensure the CI ranges between −1 and +1, the whole expression is multiplied by 2. Alternatively, it can be defined as a measure of the degree of association of between an individuals’ level of health and their relative position in the SES distribution. The negative and positive sign of CI tells us that health outcome is concentrated among poor and rich people respectively. It is important to highlight that a value of CI is equal to zero does not mean an absence of inequality, but an absence of socioeconomic gradient in the distribution, i.e. an absence of inequality associated with socioeconomic characteristics.

However, Jones and Lopez [24] illustrate that cross-sectional CIs can lead to wrong conclusions when trying to measure socioeconomic-related health inequality in the long- run as these do not take into account the possibility that people may change in socioeconomic rank. As such, they derive a formula to measure inequality in the long- run, which is similar to the cross-sectional CI. They find that the CI for the distribution of average health after T periods can be written as the difference between two terms: the weighted sum of the CIs for each of the sub periods (term1) minus a residual which is the difference between period specific SES $\left({R}_i^t\right)$ and ranks for average specific SES over all periods $\left({R}_i^T\right)$ and their relationship to health over time ( term2) as stated below in eq. 3.

3 $$ {\boldsymbol{CI}}^{\boldsymbol{T}}=\underset{\boldsymbol{T}\boldsymbol{erm}\mathbf{1}}{\underbrace{\sum \limits_{\boldsymbol{i}}{\boldsymbol{w}}_{\boldsymbol{t}}{\boldsymbol{CI}}^{\boldsymbol{t}}}}-\underset{\boldsymbol{T}\boldsymbol{erm}\mathbf{2}}{\underbrace{\frac{\mathbf{2}}{\boldsymbol{NT}{\overline{\boldsymbol{y}}}^{\boldsymbol{T}}}\sum \limits_{\boldsymbol{i}}\sum \limits_{\boldsymbol{i}}\left({\boldsymbol{y}}_{\boldsymbol{i}\boldsymbol{t}}-{\overline{\boldsymbol{y}}}^{\boldsymbol{t}}\right)\left({\boldsymbol{R}}_{\boldsymbol{i}}^{\boldsymbol{t}}-{\boldsymbol{R}}_{\boldsymbol{i}}^{\boldsymbol{T}}\right)}}\kern0.5em $$

where $\overset{=}{y}=\frac{\sum_i{\sum}_i{y}_{it}}{NT}$ is the overall average health status/population/ in T periods; $\sum \frac{{\overline{y}}_t}{T}={\overline{y}}^T$ is the average health of the individual over the T periods, ${\overline{y}}^t=\frac{\sum_i{y}_{it}}{N}$ is the mean of the health of individual in each t period, ${w}_t=\frac{{\overline{y}}_t}{T\overset{=}{y^T}}$ can be seen as the share of total health in each period; and *CI*^*T*^ is defined as long-run CI and *CI*^*t*^ is short-run CI of each health variable under consideration in period t.

The CI can be computed easily in stata software either using covariance method or regression-based method. Accordingly, this study adopts the user-written stata command conindex developed by O'Donnell et al. [34]^10^. It calculates rank-dependent inequality indices while offering a great deal of flexibility in considering measurement scale and alternative attitude to inequality. Estimation and inference is via a regression approach that allows for addressing the issue of sampling design, misspecification and for testing for differences in inequalities across population or sub-populations. The magnitude and sign of concentration index depends on the method used to compute the required index. These results also affect the inequality analysis. When the variable of interest has an infinite upper bound on a fixed scale, the main normative choice is between absolute and relative invariance. Matters are more complicated when the measurement scale is not unique. Applying the generalized CI to a ratio or cardinal variable requires one to accept that the inequality ordering may depend on the scaling adopted. This can be avoided for the relative inequality invariance criterion if one replaces the standard CI with the modified one. When the variable has a finite upper bound, one should first choose between relative inequality invariance and the mirror condition. If one prioritizes the relative invariance criterion (in attainments or shortfalls), then the standard CI or its modified version can be used. When priority is given to the mirror condition, one faces a choice between the Erreygers index, which focuses on absolute differences, and the Wagstaff index, which mixes concern for relative inequalities in attainments and relative inequalities in shortfalls [35].

In this study, for standard and generalized CI, the health variable (the dependent variable) is negative of Z-score which is continuous and unbounded variables while in case of Erreygers and Wagstaff, it is binary which is bounded variables taking a value either 1 if stunted, wasted and underweighted or 0 otherwise.

#### Mobility index and dynamics of inequality in child undernutrition

Since this study prefers to use longitudinal data, its other basic concern is examining the measurement of malnutrition inequality with variation of SES variables over time (SES related health inequality mobility). In this regard, even if individuals do not experience health changes, long-run SES- related inequality can be greater or less than that obtained with snapshot cross-sectional estimates, as long as the patterns of SES mobility are systematically related to health. Averaging the short-run measures of inequality will then tend to underestimate or overestimate the long-run picture. However, in situations where SES- related inequality tends to fade either solely due to health mobility or solely due to SES mobility, the mobility index would be zero. In these cases, the information obtained from the series of cross-sectional CIs would be sufficient to capture the dynamics of interest. Hence, it is useful to measure how much the longitudinal perspective alters the picture that would emerge from a series of cross-sections, in the same spirit as Shorrocks’ [36] index of income mobility. With same notational representation used above for computing long-run CI, Jones and Lopez [24] put mobility index M^T^ for any SES variables:

4 $$ {\boldsymbol{M}}^{\boldsymbol{T}}=1-\frac{{\boldsymbol{CI}}^{\boldsymbol{T}}}{\sum_{\boldsymbol{t}}{\boldsymbol{w}}_{\boldsymbol{t}}{\boldsymbol{CI}}^{\boldsymbol{t}}}=\frac{2}{\boldsymbol{N}{\sum}_{\boldsymbol{t}}{\overline{\boldsymbol{y}}}^{\boldsymbol{t}}{\boldsymbol{CI}}^{\boldsymbol{t}}}\left(\sum \limits_{\boldsymbol{i}}\sum \limits_{\boldsymbol{t}}\left({\boldsymbol{y}}_{\boldsymbol{i}\boldsymbol{t}}-{\overline{\boldsymbol{y}}}^{\boldsymbol{t}}\right)\left({\boldsymbol{R}}_{\boldsymbol{i}}^{\boldsymbol{t}}-{\boldsymbol{R}}_{\boldsymbol{i}}^{\boldsymbol{T}}\right)\right) $$

Here, mobility index would be different from zero if the following two conditions hold: i) The SES rank of individuals is sensitive to the length of the time window over which measurement is taken, i.e. there is SES mobility, as defined by Shorrocks [36]^11^. ii) These changes in SES rank are associated with systematic differences in health variable considered. If mobility index is negative in sign, it implies that short-run CI (cross-sectional) underestimates long-run one (longitudinal data) while it is positive, it shows that short-run CI overestimate long-run one.

Jones and Lopez [24] provide an index that measures the difference between short-run and long-run income-related health inequality and suggest that it can be interpreted as an index of health-related income mobility. Nonetheless, as of Allanson et al. [25], it is questionable whether this index is more appropriate to health policy makers other than to illustrate that income-related health inequalities may be slightly more important than might be inferred from cross-sectional estimates. Moreover, they note that, initially, health policy-makers are more likely to be interested in income-related health changes, less so in health-related income changes, especially since a large amount of health-related income changes are likely to be unavoidable.

Jones and Lopez [24] measure is equal zero if there is no income mobility regardless of whether there is health mobility. Conversely, the measure may not equal zero even if there are no health changes. Second, the index provided by Jones and Lopez [24] is symmetric in the sense that the value of the index is invariant to the ordering of the years. Yet, policy makers may want to distinguish between equalizing and dis-equalizing income changes since these have diametrically opposed implications for the level of income-related health inequality over time. Finally, the value of the Jones and Lopez [24] index is likely to be little more than a reflection of the unimodal shape of the income distribution and the strength of the association between income and health in the long- run compared to the short-run.

As a remedy for these shortcomings, Allanson et al. [25] propose an alternative approach based on the simple observation that any change in income-related health inequality over time must arise from some combination of changes in health outcomes and income ranks. By decomposing the change in between two periods, they provide an index of income-related health mobility that captures the effect on short-run income-related health inequality of differences in relative health changes between individuals with different initial levels of income. Thus, the measure addresses the question of whether the pattern of health changes is biased in favour of those with initially high or low incomes, providing a natural counterpart to measures of income-related health inequality that address the issue of whether those with better health tend to be the rich or poor. In addition, like Jones and Lopez [24], they also obtain a health-related income mobility index that captures the effect of the reshuffling of individuals within the income distribution on cross-sectional socioeconomic inequalities in health. Accordingly, in this study, Allanson et al. [25] approach is adopted to decompose the change in the short-run CI between any initial or start period s and any final period f into two part:

5 $$ {\displaystyle \begin{array}{c}{CI}^f-{CI}^s=\frac{2}{{\overline{y}}^f}\mathit{\operatorname{cov}}\left({y}_{if},{R}_{if}\right)-\frac{2}{{\overline{y}}^s}\mathit{\operatorname{cov}}\left({y}_{is},{R}_{is}\right);s,f=1,..T;s\le f\\ {}=\left(\frac{2}{{\overline{y}}^f}\mathit{\operatorname{cov}}\left({y}_{if},{R}_{if}\right)-\frac{2}{{\overline{y}}^s}\mathit{\operatorname{cov}}\left({y}_{if},{R}_{is}\right)\right)+\left(\frac{2}{{\overline{y}}^f}\mathit{\operatorname{cov}}\left({y}_{if},{R}_{is}\right)-\frac{2}{{\overline{y}}^s}\mathit{\operatorname{cov}}\left({y}_{is},{R}_{is}\right)\right)\\ {}=\left({CI}^{ff}-{CI}^{fs}\right)+\left({CI}^{fs}-{CI}^{ss}\right)={M}^R-{M}^{H\kern1.5em }\end{array}} $$

Where y_is_ and R_is_ are health and relative fractional rank of individual at starting period. Similarly, y_if_ and R_if_ denote health and relative fractional rank of individual at final period. y^f^ and y^s^ represent mean of health at final and starting period respectively. CI^ss^ and CI^ff^ are the CI ′ s in periods starting (s) and final (f) respectively, and CI^fs^ is the CI obtained when health outcomes in the final period are ranked by income in the initial period.

In equation 5, the mobility index, M^H^ = CI^fs^ − CI^ss^ provides a measure of income-related health mobility, which captures the effect of differences in relative health changes between individuals with different initial levels of income. M^H^ is positive (negative) if health changes are progressive (regressive) in the sense that the poorest individuals either enjoy a larger (smaller) share of total health gains or suffer a smaller (larger) share of total health losses compared to their initial share of health ,and equals zero if relative health changes are independent of income. M^H^ in turn depends on the level of progressivity and scale of health changes.

However, the income-related health mobility index, M^H^ is not exactly equal the change in income-related health inequality because it does not allow for the effect of changes in the ranking of individuals in the income distribution between the initial and final periods. This effect is captured by the health-related income mobility index, M^R^ = CI^ff^ − CI^fs^. It may be negative since the concentration index of final period health outcomes ranked by initial income can exceed that ranked by final income. M^R^ can be equal to zero, irrespective of the degree of reshuffling of individuals in the income distribution, if final period health is uncorrelated with changes in income rank [25].

### Measurement of inequality using decomposition method

In this part of the study, the CI of each child undernutrition indicator is decomposed in order to identify the major contributing factors to the inequality. Such decomposition method enables us to know what extent of inequality in child malnutrition is explained by inequalities in socioeconomic status such as education, health access to maternal and child health care, etc? Wagstaff, van Doorslaer, and Watanabe (2003) demonstrate that the health CI can be decomposed into the contributions of individual factors to income-related health inequality, in which each contribution is the product of the sensitivity of heath with respect to that factor (the elasticity) and the degree of income-related inequality in that factor (the respective CI).

To explain variations in a child's under-nutrition level, a standard household production-type anthropometric regression framework [37, 38] is adopted , in which negative of each child's anthropometrics indicators (Z-score) is specified to be a linear function of a vector of child-level variables, a vector of household-level variables, and community level. The study interprets this estimating equation as a reduced-form demand equation rather than a production function.

Here, the study focuses on inequalities in all malnutrition indicators measured using the negative of the child’s height-for-age z-score, weight-for-height z-score, and weight-for -age z-score respectively following the World Health Organization, WHO [32] child growth standard data. Like Wagsta et al. [27] and many others in the literature, it has two reasons for favouring the z-score over a binary variable indicating whether or not the child in question was undernutritioned or not. First, it conveys information on the depth of malnutrition rather than simply whether or not a child was malnourished. Second, it is amenable to linear regression analysis, which is favourable to the decomposition method employed in this study. Since the equation used for decomposing the CI requires linearity of the underlying regression model, most of the decomposition result holds for a linear model of health outcomes. Moreover, It uses the negative of the z-score to make the malnutrition variables easier to interpret. Rising of negative of the z-score indicates an increasing in malnutrition level. Accordingly, for its regression based -decomposition, it relies on malnutrition level rather than binary outcome as dependent variable.

Since this study employs longitudinal data, the specification of its model for decomposing socioeconomic related inequality in health could be simple pooled OLS model, random effect model and fixed effect model. Most studies in this topic use simple pooled linear model, estimating by ordinary least square (OLS) but it doesn’t take in to account potential error components structure and dynamics. This study rather uses both random and fixed effect to model and estimate the regression equation for decomposing inequality. It thus considers linear panel models^12^ as it is indicated in eq. 6.

6 $$ {\boldsymbol{Y}}_{\boldsymbol{ihct}}={\boldsymbol{\beta}}_0+{\boldsymbol{\beta}}_1{\left({\boldsymbol{X}}_1\right)}_{\boldsymbol{it}}+{\boldsymbol{\beta}}_2{\left({\boldsymbol{X}}_2\right)}_{\boldsymbol{it}}+{\boldsymbol{\beta}}_3{\left({\boldsymbol{X}}_3\right)}_{\boldsymbol{it}}+{\boldsymbol{\mu}}_{\boldsymbol{ihct}} $$

Where *Y*_*ihct*_ indicates that malnutrition level of child i in a household h, community c and in time t, X_1_, X_2_, and X_3_ are vectors of child level, household level and community level explanatory variables respectively (for details on variable definition and measurement, see Table 2). While β is a vector of regression coefficients which show the effect of X on Y; *μ*_ihct_ = *α*_i_ + *ε*_ihct_, *α*_i_ 13 is individual specific effect (could be random or non- random) effect) and *ε*_ihct_ is idiosyncratic error term.

In decomposing CI, this study follows the formula proposed by Wagsta et al. [27] while linear panel data is taken in to account in this case. Then, the decomposed CI as stated in eq. 7 shows that it is equal to the weighted sum of the CIs of the K regressors:

7 $$ {CI}^T=\sum \limits_k\left(\frac{\beta_k{\overline{X}}_k}{{\overline{y}}^T}\right){CI}_K^T+\frac{GC_{\epsilon}^T}{{\overline{y}}^T}=\sum {\eta}_k{CI}_k^T+\frac{GC_{\epsilon}^T}{{\overline{y}}^T} $$

Where CI^T^ is overall long-run CI for health, ${\overline{\mathrm{y}}}^{\mathrm{T}}$ is the mean health over all periods, *β*_k_ are coefficients obtained from regression of eq. 6, ${\overline{\mathrm{X}}}_{\mathrm{k}}$ is mean of the k^th^ regressor taken over all periods, ${\mathrm{CI}}_{\mathrm{k}}^{\mathrm{T}}$ is the long-run CI of the k^th^ regressor and ${\mathrm{GC}}_{\epsilon}^{\mathrm{T}}$ is long-run generalized concentration index for each error term^14^ and $\left({\upeta}_{\mathrm{k}}={\beta}_{\mathrm{k}}\frac{{\overline{\mathrm{X}}}_{\mathrm{k}}}{{\overline{\mathrm{y}}}^{\mathrm{T}}}\right)$ is elasticity of health variable under consideration with respect to the explanatory variables (X_k_).

Since the main objective of decomposition analysis is to offer an explanation of socioeconomic inequality of health by including the contributions of each explanatory variable to such inequality, the product of elasticity ( k) and CI of k^*th*^ regressor (${\mathrm{CI}}_k^T$) gives us the contribution of each explanatory variables in the variation of inequality in health variables.

### Blinder -Oaxaca decomposition

It is common to raise why do gaps in health outcome exist between the poor and better-off in many countries despite health systems explicitly aimed at eliminating gap in health outcome? Hence, the Oaxaca-type decomposition [34, 39] is employed to explain the difference between two groups. Such type of decomposition explains the gap in the means of an outcome variable between two groups (For example, between the poor and the non-poor). The gap is decomposed into group differences in the magnitudes of the determinants of the outcome in question and group differences in the effects of these determinants. But, such method does not allow us to decompose inequalities in health outcome across the full distribution of SES variable, rather we simply restricted to analysis between the poor and the better-off. The decomposition equation this study uses to estimate the health outcome gap between two groups is given in eq. 9. However, it takes panel data rather than different cross-sectional data for our estimate.

8 $$ {Y}_{ihct}={\beta}^P{X}_{ihct}+{\varepsilon}_{ihct}^P\kern0.75em if\kern0.5em poor $$

9 $$ {Y}_{ihct}={\beta}^R{X}_{ihct}+{\varepsilon}_{ihct}^R\kern0.75em if\kern0.5em Rich $$

10 $$ {\overline{Y}}_R-{\overline{Y}}_P=\left({\overline{X}}_R-{\overline{X}}_P\right){\beta}^P+\left({\beta}_R-{\beta}_p\right){\overline{X}}_R $$

11 $$ {\overline{Y}}_P-{\overline{Y}}_R=\left({\overline{X}}_R-{\overline{X}}_P\right){\beta}^P+\left({\beta}_R-{\beta}_P\right){\overline{X}}_R $$

where *Y*_*it*_ is individual child undernutrition level at time t, *X*_*ihc* t_ is vector of child, household and community level characteristics at time t. $\overline{X}$ represents mean of individual child undernutrition level for each group and $\overline{X}$ represents vector of child, household and community level characteristics evaluated at mean for each groups and *β*^′*s*^ also represents estimated coefficients including intercepts for poor and non-poor . So, the gap in Y between the poor and the non-poor might come from differences in the coefficients (*β*) including intercepts (difference in effects), and differences in those determinants level (X). Estimates of the difference in the gap in mean outcomes can be obtained by substituting sample means of the X ′ s and estimates of the parameter’s into eq. 8. As it is stated in eq. 12, the mean health outcome difference between the two considered gaps can be attributable to (i) differences in the X ′ s (sometimes called the explained component); (ii) differences in the β ‘s (sometimes called the unexplained component) and interaction effect (change in product of X and; β, βX).

12 $$ {\overline{\boldsymbol{Y}}}_{\boldsymbol{R}}-{\overline{\boldsymbol{Y}}}_{\boldsymbol{P}}=\left({\overline{\boldsymbol{X}}}_{\boldsymbol{R}}-{\overline{\boldsymbol{X}}}_{\boldsymbol{P}}\right){\boldsymbol{\beta}}^{\boldsymbol{P}}-\left({\boldsymbol{\beta}}_{\boldsymbol{R}}-{\boldsymbol{\beta}}_{\boldsymbol{P}}\right){\overline{\boldsymbol{X}}}_{\boldsymbol{R}}\kern0.5em +\left({\overline{\boldsymbol{X}}}_{\boldsymbol{R}}-{\overline{\boldsymbol{X}}}_{\boldsymbol{P}}\right)\left({\boldsymbol{\beta}}_{\boldsymbol{R}}-{\boldsymbol{\beta}}_{\boldsymbol{P}}\right) $$

## Results and discussion

This part is basically devoted for result interpretation and analysis on inequalities in malnutrition based on different approach of measuring inequality and its dynamics. It also covers analysis on contribution of major factors incorporated for the inequalities prevalence using decomposition method.

### Basic descriptive statistics

**Fig. 1:**
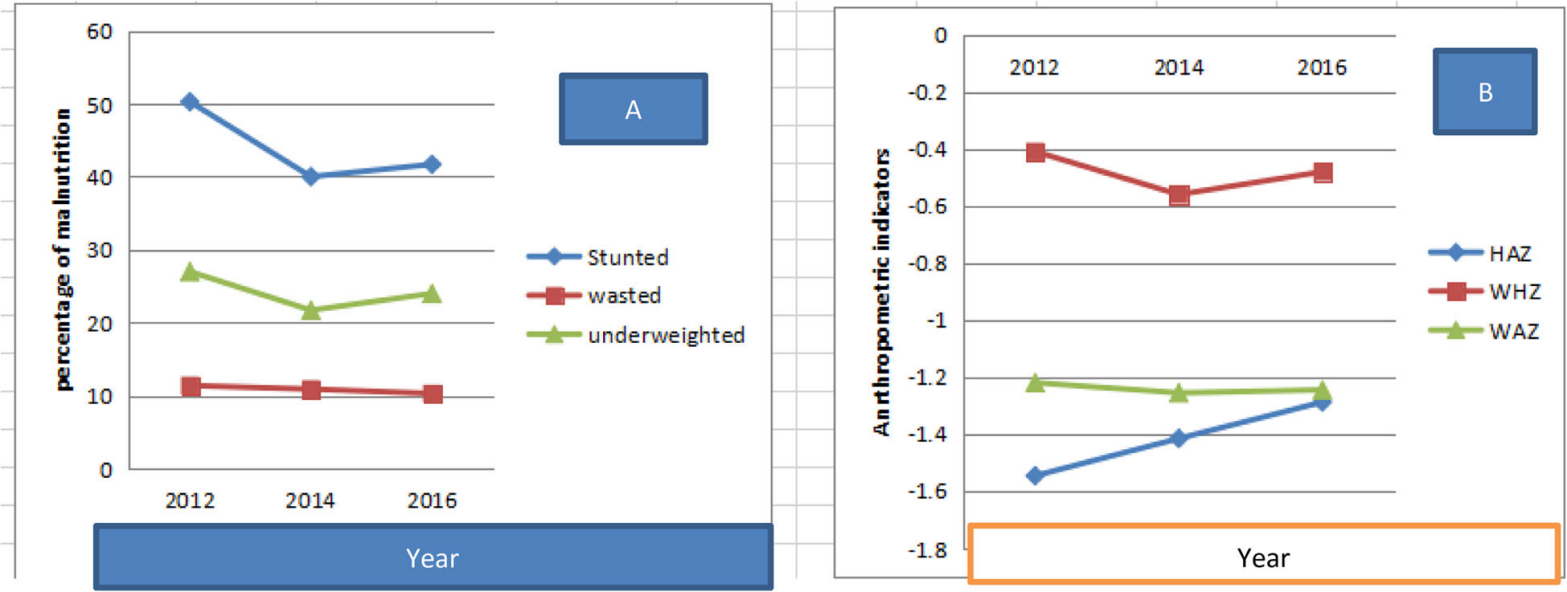
Trend of malnutrition and anthropometric indicators across wave

It is noteworthy to see first some basic descriptive statistics on major health and socioeconomic variables used in this study. Referring to Fig. 1, from 2011/12 to 2015/16, one can observe that percentage of undernutritioned children in all indicators (on average) falls.

As it is aforementioned, the final data used in this study is constructed from various individual, household and community level covered in all three survey waves. The health variable data is prepared from each individual’s child’s age, sex, weight, and height, using Zanthro ado file with reference to World Health Organization, WHO [32] child growth standards.

As it is shown in Table 3, finally, total of 11,061 individual observations from those three waves are considered for this study’s analysis. However, it uses a balanced panel data with observations of 6087 individuals for measuring dynamic of inequalities over time using mobility indices. Then, outliers and normality tests are conducted for major socioeconomic variables.

**Table 3:** Summary statistics of variables used in regression for decomposition

Variable	Obs	Mean	Std. Dev
Age_months	11061	45.73339	27.44576
Agesqr	11061	2844.744	2829.643
llness incidence	10835	.1728657	.3781486
Water_availability	11049	.4469679	.2758621
Toilet facility	11056	6.342438	1.205653
Health post	10819	1.105093	.3066873
mother education	10767	.4592737	.9098248
Household size	11061	6.23063	2.020077
Household size under 5 age	11061	1.504114	.8234286
HAZ	9011	−1.3873	1.73204
WHZ	8415	−.49157	1.43958
WAZ	9784	−1.24230	1.30505
Wealth index	11007	−.7662025	1.444134
Consumption per capita	10785	5278.117	4394.013

Figure 3 shows an overview of distribution of child malnutrition indicators by their Z-score. Similarly, Fig. 4 signifies that the distribution of wealth index is more concentrated to the left with negative sign which indicates that most of the households are poor. It also apparently shows that real annual consumption per adult equivalent is skewed to the right for the clear reason that consumption can’t be negative in values (see Figure 3 and 4 in the appendix).

Basically, the analysis of anthropometric data is used for the identification of undernourishment in a population or sub-population. Accordingly, a first step is to look at the distribution of the z-scores and the overall prevalence of undernourishment. When compared with the distribution of z-scores in the reference population, this provides a first impression of different dimensions of nutritional status in the population.

As it is displayed at Fig. 3, almost in all Z-scores, the distribution is skewed to the left which implies that many individuals are away from the median of the distribution. HAZ-score and WAZ-score are also positively correlated while HAZ and WHZ-score are negatively correlated.

### Inequality in undernutrition

Before measuring inequality using complex approach, it is common to use simple approach which is helpful merely to look at the absolute mean difference of anthropometric score between two groups. In due respect, significant mean difference is exhibited between different groups considered in this analysis such as rural and small town, bottom 60% and top 40%, richest and poorest, male and female. This shows that the prevalence of malnutrition is disproportionately distributed across different groups (for details, see Table 4).

**Table 4:** Mean difference of anthropometric indicator between two groups

Groups	HAZ	WHZ	WAZ
Small town	−1.1(.064)	−.33 (.057)	−.84 (.048)
Rural	−1.4(.018)	−.50 (.016)	− 1.2 (.013)
Deference (Small town -Rural)	.30** (.070)	.16*** (.061)	.42*** (.051)
Male	− 1.4(.025)	−.47 (.022)	− 1.2 (.018)
Female	−1.3 (.026)	−.51 (.022)	−1.1. (018)
Deference (Male -Female)	−.06*(.036)	.04(.031)	−.08*** (.026)
Wealth index			
_Poorest(1_st quintile)	−1.5 (.062)	−.57 (.054)	−1.4 (.042)
_Richest (5_thquintile)	−1.1 (.079)	−.26 (.067)	−.86 (.058)
Difference (1st-5th^)^	−.44*** (.103)	−.31** (.089)	−.59*** (.072)
Non-poor(Top 40%)	−1.1(.031)	−.42 (.027)	−1.03 (.023)
Poor(Bottom 60%)	−1.4 (.022)	−.52 (.019)	− 1.33 (.015)
Difference (40–60%)	.30*** (.039)	.09*** (.033)	.29*** (.028)
Consumption			
_Poorest(1_stquintile)	−1.5 (.065)	−.61 (.056)	−1.4 (.045)
_Richest (5_thquintile)	−1.2 (.075)	−.27 (.064)	−.94 (.053)
Difference (1^st^-5^th)^	−.35** (.099)	−.34** (.085)	−.53*** (.069)
Non-poor (Top 40%)	−1.2 (.030)	−.45 (.026)	−1.1 (.021)
Poor(Bottom 60%)	−1.4 (.023)	−.51 (.019)	− 1.3 (.016)
Difference (40–60%)	.22*** (.038)	.06* (.032)	.20*** (.027)

Significance level ***, ** and * is at 1%, 5 and 10% respectively and; Std. Errors are in parenthesis. Two-sample t test with equal variances (Ho: difference is zero; H1: difference is different from zero

In terms of HAZ- malnutrition level, regions can be ranked from highest to lowest as Tigray, Amhara, SNNP, Oromia, and Other regions respectively while in WHZ- malnutrition level, it is as follows Tigray, Other regions, Amhara, Oromia, and SNNP respectively. Similarly, with WAZ- malnutrition level, it is given as Tigray, Amhara, Other regions, SNNP, and Oromia respectively (for details, see Table 17, in the appendix part).

Since pairwise comparisons ignore all other subgroups that are not being compared, it is common to employ multiple (complex) measures in the analysis of inequality. The most common and appropriate methods for measuring inequality magnitude and directions are thus concentration curves and index.

**Fig. 2:**
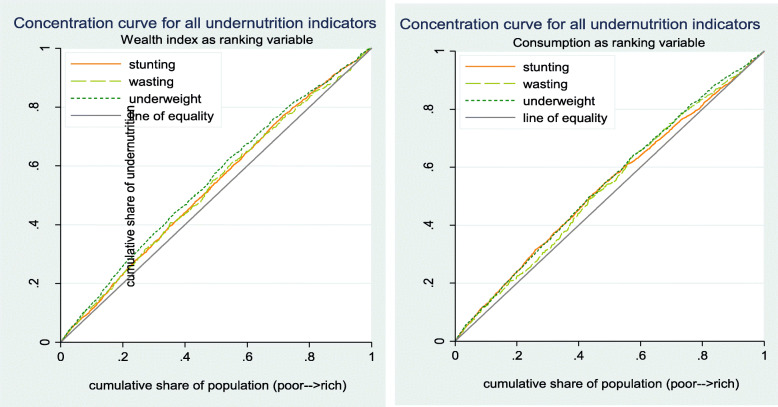
Concentration Curves of Undernutrition Indicators

**Fig. 3:**
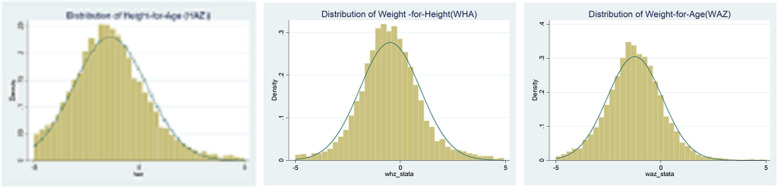
Distribution of Anthropometric Indicators

**Fig. 4:**
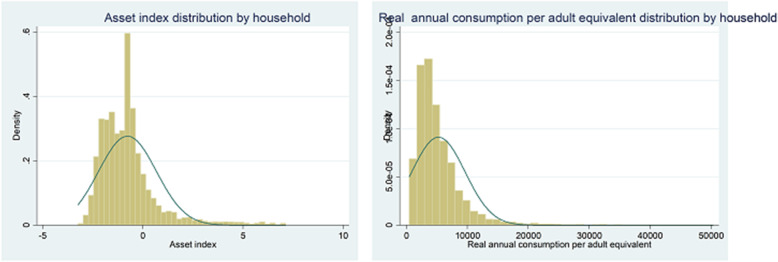
Socioeconomic distribution by household (normalized)

As it is illustrated in Fig. 2, the concentration curves for each undernutrition indicators is located above the line of equality. These indicate that higher malnutrition level is disproportionately prevailed among the poor section of the population in both SES ranking variables, i.e. pro- poor inequality in terms of malnutrition level.

Estimation and inference is via a regression approach, user-written stata command conindex, developed by O'Donnell et al. [35] that allows for addressing the issue of sampling design, misspecification and for testing for differences in inequalities across population or sub-populations. For standard and generalized CI, the health variable is negative of Z-score which is continuous and unbounded variables while in case of Erreygers and Wagstaff, it is binary which is bounded variables (either 0 or 1).

As it is shown in Table 5, the concentration indices for each malnutrition indicators and SES ranking variables vary across the methods employed for computing those indices. In all approaches and SES ranking variables, the CIs are significant with negative value which exhibit higher malnutrition in all indicators is disproportionately observed in poor part of the population. While employing different SES ranking variables, the difference in the concentration indices is only found significant in case of Height-for-age Z-score (HAZ). Using standard method, for example, in HAZ, -0.040 and -0.070 of CI for wealth index and consumption are scored respectively. It signifies that relatively higher inequality is measured using consumption as SES ranking variable.

**Table 5:** Concentration indices (CI) of malnutrition prevalence by methods: Ranking variables -wealth index and consumption

Method/Indicators	Standard CI	Consumption	CIc-CIw	Generalized CI	Consumption	CIc-CIw
	Wealth		Difference	Wealth		Difference
HAZ	−.040*** (.011)	−.070*** (.012)	−.024** (.011)	−.065*** (.019)	−.114*** (.020)	−.039** (.017)
WHZ	−.028** (.012)	−.023*** (.013)	.000 (.012)	−.023** (.010)	−.019* (.011)	.000 (.010)
WAZ	−.061*** (.010)	−.059*** (.010)	.002 (.009)	−.087*** (.014)	−.084*** (.015)	.004 (.015)
Observation	6087	6087	5133	6087	687	5133
	Erreygers-	Normalized		Wagstaff-	Normalized	
Stunting	−.093*** (.024)	−.111*** (.025)	−.054** (.023)	−.107*** (.028)	−.132*** (.029)	−.059** (.025)
Wasting	−.028** (.012)	−.031*** (.012)	−.009 (.014)	−.083** (.036)	−.092*** (.035)	−.025 (.037)
Underweight	−.132*** (.022)	−.108*** (.021)	.007 (.025)	−.172*** (.029)	−.140*** (.024)	.010 (.032)
Observation	6087	6087	5133	6087	6087	5133

Significance level ***, ** and * is at 1%, 5 and 10% respectively and; Std. Errors(in parenthesis) are adjusted for each clusters in ea_id (enumeration areas or primary sampling units)

Using Wagstaff method, for example, in stunting, − 0.093 and − 0.111 of CI for wealth index and real annual total consumption per adult equivalence are observed respectively. With the same method, in terms of SES ranking variables altering, the highest CI and thus inequality, in each malnutrition indicators is relatively recorded in case of wealth index. From these results, one can thus infer that in all SES ranking variables, higher inequality of malnutrition is concentrated in poor part of the society.

Another concern of this study is examining malnutrition inequalities using spatial dimensions and across other groups considered in this analysis. For each malnutrition indicators, CI is computed for each regions, male-female, rural-urban and then compares them to see the existence of significant difference between those groups considered. Thus, this study’s results signify that significant inequality of malnutrition difference is shown across regions. It also recognizes same result across lower administrative areas such as provinces (Zones), districts (Woredas) and Kebeles (lowest administrative units). For instance, in Height-for-Age Z-score (HAZ) with wealth index as ranking variable, the highest and lowest inequality of malnutrition is seen in SNNP (CI = -0.054) and Tigray (CI = -0.029) regions respectively. However, when real consumption per adult equivalence is taken in to account as ranking variable, the highest and lowest malnutrition inequality is observed in SNNP and Other regions respectively. As it is displayed in Table 6, in case of the other malnutrition indicators such as Weight-for-Height Z-score (WHZ) and Weight-for-Age Z-score (WAZ), analysis of inequality could be different. In terms of sex-wise, except in consumption as ranking variables for WHZ and WAZ, the difference is insignificant. Similarly, inequality difference is almost insignificant while rural-urban is considered. In short, regardless of its significance, malnutrition inequality varies across considered groups in each indicator while ranking SES variables is altered (for details, see Table 7)^15^.

**Table 6:** Concentration indices of malnutrition prevalence by region: Ranking variables -wealth index and consumption

Regions	Height-for-Age	(HAZ)	Weight-for-Height	(WHZ)	Weight-for-Age	(WAZ)
	Wealth	Consumption	Wealth	Consumption	Wealth	Consumption
Tigray	−.029** (.021)	−.053** (.022)	−.001 (.025)	.021 (.042)	−.050*** (.016)	−.038*
						−.017
Amhara	−.036* (.023)	−.019* (.014)	−.069*** (.025)	−.036 (.025)	−.052** (.021)	−.017 (.013)
Oromia	−.035** (.015)	−.039** (.016)	−.036* (.022)	−.028 (.021)	−.047*** (.013)	−.040** (.016)
SNNP	−.054*** (.010)	−.057*** (.019)	−.010 (.020)	−.038 (.028)	−.057*** (.013)	−.067** (.020)
Other	−.052**	−.017	−.040	.006	−.053***	−.015
Regions	(.023)	(.023)	.028	(.019)	(.016)	(.015)
Difference	1%	1%	1%	1%	1%	1%

Significance level: ***, ** and * is at 1%, 5 and 10% respectively; and Std. Errors(in parenthesis) are adjusted for each clusters in ea_id (enumeration areas or primary sampling units)

**Table 7:** Concentration indices of malnutrition prevalence by sex and rural small town: Ranking variables -wealth index and consumption

Groups	Height-for-Age	(HAZ) Weight-for-Height		(WHZ) Weight-for-Age (WAZ)		
	Wealth	Consumption	Wealth	Consumption	Wealth	Consumption
Male	−.044*** (.011)	−.051*** (.012)	−.041** (.014)	−.038** (.015)	−.061*** (.010)	−.051*** (.011)
Female	.049*** (.011)	−.044*** (.011)	−.021 (.015)	.018 (.016)	−.047*** (.011)	−.023* (.012)
Difference	not sign	not sign	not sign	5%	not sign	5%
Small town	−.090** (.034)	−.002 (.034)	.024 (.052)	.009 (.045)	−.073* (.044)	−.026 (.032)
Rural	−.043*** (.009)	−.048*** (.009)	−.031*** (.012)	−.019 (.013)	−.049*** (.009)	−.044*** (.009)
Difference	not sign	5%	not sign	no sign	no sign	no sign

Significance level: ***, ** and * is at 1%, 5 and 10% respectively; and Std. Errors (in parenthesis) are adjusted for each clusters in ea_id (enumeration areas or primary sampling units)

### Mobility indices and SES-related inequality in children undernutrition

**Fig. 5:**
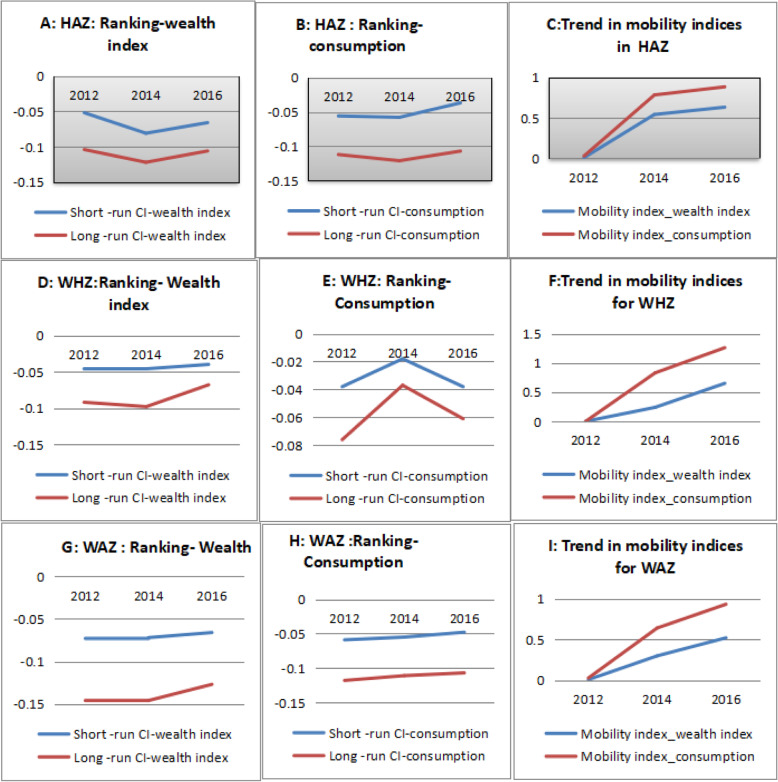
Trends in short-run and long-run concentration indices, and mobility indices

The basic argument here is that taking on CI of each cross-sectional data or weighted average of them hides the effect of time on inequality and fail to see dynamics of SES related inequality. It is either by the short-run CI underestimates or overestimates the long-run CI. This again leads to wrong inequality measurement inference. As it can be discerned from Fig. 5 in the appendix, there is apparent trends in short-run and long-run CIs in all undernutrtion indicators and SES ranking variables. This is a clear indication for existence of health -related SES mobility indices.

Results from Table 8 show us that in both malnutrition indicators and SES ranking variables, the mobility indices are positive which implies that short-run (cross-sectional) CI overestimates the long-run (longitudinal data) CI. Hence, the results exhibit that the long-run SES related inequality in malnutrition declines while longitudinal data is considered, rather than using the weighted average of the cross-sectional CIs. For example, in case of Height-for-age Z-score (HAZ) with wealth index as ranking variable, the mobility index is 0.54 and 0.63 for second and third wave respectively. It can be interpreted as the short-run measure overestimates long-run pro-poor inequality by 54 and 63% over respected waves for HAZ -malnutrition with wealth index as ranking variable i.e., SES related inequality in HAZ -malnutrition decreases by 54 and 63% over respected waves.

**Table 8:** Concentration and mobility indices for each undernutrition indicators: Ranking variables -wealth index and consumption

Wave	Wealth			Consumption		
	CI^**t**^	CI^**T**^	M^**T**^	CI^**t**^	CI^**T**^	M^**T**^
**Height-for-Age**	**Z-score**					
2011/12	−.052	−.052	0	−.056	−.056	0
2013/14	−.080	−.041	.54	−.058	−.063	.24
2015/16	−.066	−.040	.63	−.037	−.070	.25
**Weight-for-Height**	**Z-score**					
2011/12	−.046	−.046	0	−.038	−.038	0
2013/14	−.046	−.052	.24	−.018	−.019	.59
2015/16	−.040	−.028	.65	−.038	−.023	.61
**Weight-for-Age**	**Z-score**					
2011/12	−.073	−.073	0	−.059	−.059	0
2013/14	−.072	−.074	.30	−.055	−.056	.34
2015/16	−.066	−.061	.52	−.048	−.059	.41

CI^t^ is CI at time t (each wave) or short-run CI and CI^T^ is long-run CI (for longitudinal data). M^T^ is mobility index for each wave

If M^T^ > 0, CI^t^ overestimates CI^T^ while M^T^ < 0, CI^t^ underestimates CI^T^; and M^T^_=0_^,^ no change in inequality

Similarly, for real annual consumption per adult equivalent as ranking variable, it makes long-run SES-related health inequality greater than what we could infer from the cross-sectional measures or it declines by 24 and 25%, as reflected by the mobility index (M^T^) of 0.24 and 0.25 in second and third wave respectively. These results and analyses strengthen this study’s initial argument that examining SES related inequality using cross-sectional data masks the effect of dynamics on inequality over time (fails to see the correct long-run CI and thereby inequality). In general, Table 8 illustrates that the health-related income mobility index and shows that, by the last (third) wave, the short-run measure over estimates long-run inequality by around 63 and 25%, 65 and 61%, and 52 and 41% for HAZ, WHZ and WAZ respectively while wealth index and consumption are considered as ranking variable. Therefore, employing longitudinal perspective rather than weighted average of cross-sectional data is justifiable to see the dynamic of inequality in child malnutrition. However, Allanson et al. [25] question the value of the Jones and Lopez [24] index to health policymakers and proposes an alternative index of income-related health mobility, based on a decomposition of the change in the short-run concentration index over time, that measures whether the pattern of health changes is biased in favour of those with initially high or low incomes.

Based on Allanson et al. [25] approach, the decomposition of change in inequality (CI) between wave 1 and each subsequent wave, as illustrated in Table 9 provides us both SES-related health mobility and health-related SES mobility indices. Sign of the index of SES-related health mobility, M^H^ is both positive and negative for given time spans, in each malnutrition indicator. When it is positive, it implies that differences in relative health changes experienced on average by individuals with different initial levels of SES had the effect of reducing socioeconomic inequalities in health. While, negative sign of M^H^ has regressive effect which indicates that differences in relative health changes had the effect of rising socioeconomic inequalities in health. Put it differently, when decomposing the initial and final CIs, health changes are found to be biased against those in the lower (upper) end of the SES rankings as the SES-related health mobility index is negative (positive) respectively.

**Table 9:**
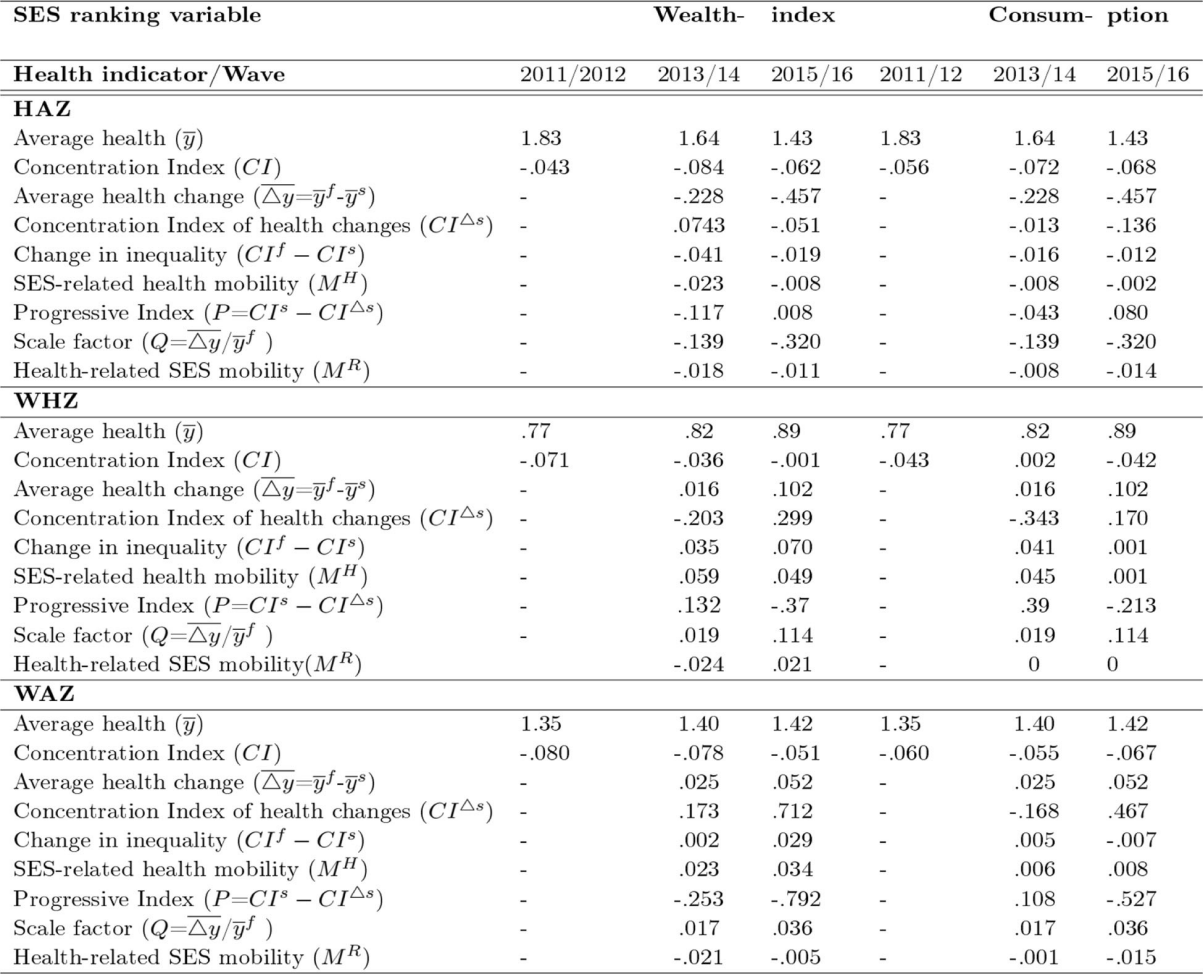
SES-related health mobility and Health-related SES mobility index from Wave 1 (2011/12), based on Allanson et al. [25] approach

Note: *M*^*H*^=*CI*^*fs*^*-CI*^*ss*^*.CI*^*ss*^ and *CI*^*ff*^ are the CI’s in periods *s* and *f* respectively, and *CI*^*fs*^ is the CI obtained when health outcomes in the final period are ranked by SES in the initial period. *M*^*R*^ *= CI*^*ff*^ *–CI*^*fs*^*.CI*^*ff*^ and *CI*^*fs*^ are the CI’s in periods *f*, and *CI*^*fs*^ CI obtained when health outcomes in the final period are ranked by SES in the initial period. *CI*^△*s*^ represents mean health change ranked by initial rank (the concentration coefficient of health changes ranked by initial period income)

Similarly, the sign of health related SES mobility index, M^R^ is mixed. Positive sign indicates that those who moved up the income ranking tended to be healthier in the final period compared to those who moved down. And the reverse is true while it bears negative sign. In other words, the positive/negative/ values on the health-related SES mobility index suggest that the healthy are more upward/downward/ mobile respectively.

Specifically, in case of HAZ, the sign of both SES related health mobility index (M^H^) and health related SES mobility index _(_M^R^) are negative in both wealth index and consumption. It implies that individuals face regressive effect (M^H^ < 0) from health change as well as progressive effect from SES ranking change (M^R^ < 0) and the counter balance effect leads to a cumulative effect of no change in inequality change. In other word, persistence of SES inequality in HAZ occurs in the long-run. This result doesn’t confirm results obtained from mobility indices computed based on the Jones and Lopez [24] approach. Similarly, results on WAZ show that M^H^ > 0 and M^R^ < 0. This indicates that individuals face progressive effect in both indices. Thus, it has a cumulative effect of reducing effect on inequality in the long-run which confirms results that are obtained based on Jones and Lopez [24] approach. However, for WHZ (short -run indicator), there is no clear trend over subsequent waves to put any concluding remarks.

### Decomposing inequality of undernutrition

Since the eq. (6) used for decomposing the CI requires linearity of the underlying regression model, for this study’s decomposition tasks, negative of each child Z-score (as malnutrition level which is continuous variable against the relevant covariates^16^) is employed. The regression results in random as well as other alternative estimator are given in the appendix, Tables 18 and 19. The coefficients are presented along with robust standard errors that are adjusted for clustering to enumeration areas (primary sampling units) due to the use of panel survey data. Decomposition results based on the alternative estimator, fixed effect is also attached at the appendix part, Tables 18 and 19^17^.

Each column under each malnutrition indicators in Tables 10 and 11 presents elasticity of each regressor with respect to the health variable considered, CI of each regressor, contributions to the overall CI as well as percentages contribution of the overall CI which is given in the parenthesis.

**Table 10:** Decomposition of Child Malnutrition Inequality (CI): Ranking Variable Wealth Index

Regressors (k)	*β*_*k*_	HAZ				WHZ				WAZ		
		Elasticity	CI	Contribution	*β*_k_	Elasticity	CI	Contribution	*β*_k_	Elasticity	CI	Contribution
Age	.006* (.002)	.17	−.03	−.01(.11)	−.014***(.002)	−.81	−.03	.02(−.72)	.007*** (.002)	.24	−.03	.01(.12)
Age-square	−.001* (.000)	−.18	−.05	.01(−.20)	−.000(.000)	.50	−.05	−.03(.82)	.000 (.000)	−.06	−.05	.00(−.05)
Sex	.077** (.035)	.02	.02	.00(−.01)	−.018(.025)	−.01	.02	−.00(.01)	.089***(.025)	.03	.02	.00(−.01)
Illness incidence	.103** (.040)	.01	.05	.00(−.01)	.107*** (.032)	.02	.06	.00(−.03)	.180***(.030)	.02	.05	.00(−.02)
Water availability	−.057 (.055)	−.01	.19	−.00(.03)	.045 (.038)	.01	.19	.00(−07)	.008(.043)	.00	.19	.00(−.00)
Toilet type	−.002 (−.016)	−.01	−.02	.00(−.00)	.034***(.013)	.26	−.02	−.01(.17)	.037***(.013)	.16	−.02	−.00(.07)
Health post	−.141** (.067)	−.09	−.00	.00(−.00)	.011 (.058)	.01	.00	4.3(−.00)	−.078(.058)	−.06	−.00	.00(−.00)
Mother educ	–	−.03	.32	−.01(.20)	−.024 (.015)	−.01	.31	−.00(.11)	−.074***(.018)	−.02	.32	−.01(.13)
	.110***(.022)											
Household size	−.016 (.010)	−.06	.01	−.00(.01)	.001 (.008)	.01	.01	.00(−.00)	−.006(.008)	−.03	.01	−.00(.00)
Household sizeU5	−.039(.025)	−.03	−.01	.00(−.00)	−.001 (.018)	−00	−.00	4.6(−.00)	−.049**(.020)	−.05	−.00	.00(−.00)
Rural-urban	yes	.02	−.01	−.00(.01)	yes	.13	−.01	−.00(.05)	yes	.16	−.01	−.00(.04)
Region variation	yes			−.003(.08)	yes			.00(.-12)	yes			−.00(.03)
Wealth index				−.01(.30)	−.039***(.011			−.03(.91)	−.045***(.013)			−.02(.30)
	−.045***(.016)											
Quntile 1		.02	−.80	−.02(.34)		.03	−.80	−.02(.70)		.02	−.80	−.02(.30)
Quntile 2		.01	−.40	−.01(.12)		.01	−.39	−.01(.17)		.01	−.40	−.00(.06)
Quntile 3		.01	.02	.00(−.00)		.01	.01	.00(−.00)		.01	.01	.00(−.00)
Quntile 4		.02	.42	.01(−.16)		−.00	.41	−.00(.04)		.01	.41	.00(−.06)
Residual				−.022(.45)				004(−.13)				−.021(.42)
Observation	8686				8686				9426			
R^2^	0.03				0.03				0.052			

Significance level: ***, ** and * is at 1% , 5% and 10% respectively; and Std. Errors ( in parenthesis) are adjusted for each clusters in ea_id (enumeration areas or primary sampling units). Under each malnutrition indicators, in the contribution column, the figure in parenthesis represents the percentage contribution. Each figure is rounded to two digits only. Hence, point zero zero doesn't mean that it is actually zero, it is rather rounded value

**Table 11:** Decomposition of Child Malnutrition Inequality (CI): Ranking Variable- Consumption

Regressors (k)	*β*_*k*_	HAZ				WHZ				WAZ		
		Elasticity	CI	Contribution	*β*_*k*_	Elasticity	CI	Contribution	*β*_*k*_	Elasticity	CI	Contribution
Age	.006**(.003)	.19	.01	.00(−.02)	−.015***(.002)	−.82	−.00	.00(−.15)	.008***(.002)	.27	.01	.00(−.03)
Age-square	−.000 (.000)	−18	.01	−.00(.04)	−.000(.000)	.53	−.00	−.00(.08)	−.000(.000)	−.07	.01	−.00(.02)
Sex	.080**(.035)	.02	−.00	−.00(.00)	−.016(.025)	−.01	.00	−.00(.00)	.091***(.026)	.03	.00	.00(−.00)
Illness incidence	.108***(0.041)	.01	−.00	−.00(.00)	.111***(.032)	.02	.00	.00(−.00)	.189***(.031)	.02	−.01	−.00(.00)
Water availability	−.072 (.056)	−.01	.05	−.00(.01)	.036 (.038)	.01	.04	.00(−.03)	−.003(.043)	−.00	.04	−.00(−.00)
Toilet type	.001(.017)	.00	−.00	−.00(.00)	.039***(.012)	.29	−.00	−.00(.05)	.040***(.013)	.18	−.00	−.00(.01)
Health post	−.137**	−.09	.01	−.00(.01)	.009(.061)	.01	.01	.00(.02)	−.083(.056)	−.06	.01	−.00(.01)
	(.067)											
Mother educ	−.111***	−.02	.26	−.01(.15)	−.027* (.016)	−.01	.26	−.00(.21)	−.074***(.017)	−.02	.25	−.01(.13)
	(.021)											
Household size	−.024**	−.09	−.03	.00(−.06)	−.003(.008)	−.02	−.03	.00(−.05)	−.012(.008)	−.05	−.03	.00(−.04)
	(.010)											
Household sizeU5	−.032 (.025)	−.03	−.01	.00(−.01)	.001 (.019)	.00	−.00	−.00(.00)	−.045**(.020)	−.05	−.01	.00(−.01)
Rural-urban	yes	.04	−.01	−.00(.01)	yes	.15	−.00	−.00(.05)	yes	.17	−.01	−.00(.02)
Region	yes			−.01(.12)	yes			.00(−.27)	yes			−.00(.07)
variation												
Consumption	–			−.02(.48)	−.029(.023)			−.01(.71)	−.099***(.024)			−.02(.49)
	.126***(.033)											
Quntile 1		.02	−.80	−.02(.36)		.01	−.80	−.01(.54)		.02	−.80	−.02(.42)
Quntile 2		.02	−.40	−.01(.13)		.01	−.40	−.00(.24)		.01	−.40	−.01(.13)
Quntile 3		.01	.00	.00(−.00)		.02	.01	.00(−.02)		.02	.01	.00(−00)
Quntile 4		.00	.40	.00(−.01)		.00	.41	.00(−.05)		.01	.40	.00(−.05)
Residual				−.013(.28)				−.006(.33)				−.014(.31)
Observation	8505				7973				9229			
R^2^	0.039				0.045				0.052			

Significance level: ***, ** and * is at 1% , 5% and 10% respectively; and Std. Errors ( in parenthesis) are adjusted for each clusters in ea_id (enumeration areas or primary sampling units). Under each malnutrition indicators, in the contribution column, the figure in parenthesis represents the percentage contribution. Each figure is rounded to two digits only. Hence, point zero zero doesn't mean that it is actually zero, it is rather rounded value

Comparatively, this study’s findings indicate that there is very limited contribution of the legitimate factor (such as age) in all malnutrition inequalities which signify that almost all are due to illegitimate factors such as wealth index, illness toilet facility etc. In Height-for-age Z-score (HAZ) and Weight-for-age Z-score (WAZ), wealth index and mother’s education are the major contributors of socioeconomic related inequality in children undernutrition. For example, wealth index and mother’s education contribute 30 and 20%, 91, and 11% in case of HAZ and WAZ respectively while in Weight-for-Height Z-score (WHZ), the loin share is taken by wealth index (30%) and toilet facility (17%). Of course, the contribution of unexplained (residual) of the econometric model is higher for HAZ and WAZ. It accounts 45, 13, and 42% of total contribution in case of HAZ, WHZ and WAZ respectively. The contribution of other factors such toilet facility is nil for HAZ while it is 17 and 7% for WHZ and WAZ respectively. Similarly, the contribution of sex, health facility and household size is almost zero in all malnutrition indicators. Illness incidence contributes 1, 3, and 2% in case of HAZ, WHZ, and WAZ consecutively.

The contribution of mother education varies across malnutrition indicators. It is higher (20%) in case of the long-run malnutrition indicator, low HAZ (stunting). Here, the possible reason could be due to the fact that effect of formal education is more pronounced on long-run than short -run indicator [12]. However, in case of short-run malnutrition indicator (low WHZ or wasting) and composite malnutrition indicator (low WAZ or underweight), mother education level accounts for 11 and 13% of the total contribution of observed inequalities in malnutrition.

While socioeconomic ranking variable is changed from wealth index to real annual total consumption per adult equivalent, different result is observed. As in wealth index case, results indicate that contribution of legitimate factor (such as age) is a very insignificant which signify that almost all is due to illegitimate factors such as consumption, illness toilet facility etc. In HAZ and WAZ, consumption and mother’s education represent as the major contributors of socioeconomic related inequality in children undernutrition. For example, contribution of consumption and mother’s education account for 48 and 15%, 71 and 21%, 42, and 13% in case of HAZ, WHZ, and WAZ respectively. In a similar fashion, the contribution of other factors such as toilet facility, illness, sex, water availability and health facility is almost zero in all malnutrition indicators. Household size contributes 6, 5, and 4% in case of HAZ, WHZ and WAZ consecutively. The contribution of unexplained (residual) of the econometric model also accounts for 28, 33, and 31% of total contribution in HAZ, WHZ and WAZ respectively.

In both SES ranking variables, the contribution of the residuals of the econometric model is large enough as the contributions of the regressors, an indication of the presence of systematic unobserved heterogeneity which will have to be tackled with more sophisticated econometric specifications.

In terms of related groups, the contributions of time variant factors (in all socioeconomic variables) strongly dominate that of time invariant (fixed variables like place of residence). The contribution of regional variation in both wealth index and consumption is 8 and 12%, 12 and 27%, and 3 and 7% for HAZ, WHZ and WAZ respectively. Similarly, rural-urban variation contributes 1 and 1%, 5 and 5%, 4 and 2% respectively. Though it varies from one malnutrition to other malnutrition indicator, the contribution of regional as well as rural-urban related variation to the inequality is thus smaller by large compare to socioeconomic related variation. These imply that in both SES variables, the bulk of inequality in malnutrition is caused by inequality in socioeconomic status in which it disfavors the poor in both cases (for details, see Table 12).

**Table 12:** Decomposition of Child Malnutrition Inequality (CI): Over all contribution by related groups Ranking variables -wealth index and consumption

Categories	HAZ-	Score	WHZ-	Score	WAZ-	Score
	Wealth	Consumption	Wealth	Consumption	Wealth	Consumption
Wealth/consumption	−.01(.30)	−.02(.48)	−.03(.91)	−.01(.71)	−.02(.30)	−.02(.49)
Health -care	−.001(.02)	−.00(.02)	−.00(.07)	−.00(.02)	−.00(.05)	−.00(.02)
Family size	−.00(.01)	.00(−.07)	.00(−.00)	.00(−.05)	−.00(.00)	.00(−.05)
Mother educ	−.01(.20)	−.01(.15)	−.00(.12)	−.00(.21)	−.01(.13)	−.01(.13)
Time variant	−.02(.43)	−.03(.60)	−.04(1.2)	−.01(.82)	−.03(.56)	−.03(.57)
Regional variation	−.003(.08)	−.01(.12)	.00(.-12)	.00(−.27)	−.00(.03)	−.00(.07)
Rural-urban variation	−.00(.01)	−.00(.01)	−.00(.05)	−.00(.05)	−.00(.04)	−.00(.02
Time invariant	−.003(.08)	−.01(.12)	.00(−.06)	.00(−.22)	−.00(.06)	−.00(.09)
Residual	−.022(.45)	−.013(.28)	004(−.13)	−.006(.33)	−.021(.42)	−.014(.31)

under each malnutrition indicators, in the contribution column, the figure in parenthesis represents the percentage contribution. Each figure is rounded to two digits only. Hence, point zero zero doesn’t mean that it is actually zero, it is rather rounded value

### Decomposing poor- non-poor differences in child undernutrition

Before estimate the decomposition equation, this study first tests null of no differences in mean dependent variables, covariates, and regression coefficients between the two groups while allowing sample weights and clustering. As result, significant difference in all attributes to mean outcome difference for HAZ and WAZ is observed. While the results are insignificant for WHZ. In its estimation, it considers different cases like three-fold decomposition (endowments, coefficients and interactions), two-fold decomposition (with poor or non-poor coefficients as the reference) and two-fold decomposition with pooled coefficients as the reference (with group or without group variable included in the pooled model). Coefficients, means and predictions for both poor, rich and pooled are also computed. Decomposition results that show how each covariates explain the non-poor-poor gap in undernutrition can be provided upon request.

Our Blinder-Oaxaca decomposition analysis is conducted to decompose the poor - non-poor differences in child malnutrition outcomes into two components; one that is explained by differences in the level of the determinants (covariate effects), and another component that is explained by differences in the effect of the determinants on the child nutritional status (coefficient effects).

Results on Table 15 show that the poor- non-poor gap in child malnutrition is significant in all indicators. The explained and unexplained (coefficient) effects are only significant in case of HAZ and WAZ while interaction effects are insignificant in all indicators. Other results also show that the explained (covariate) effect is dominant while the coefficients effects are relatively low in the all malnutrition indicators. SES variables such as wealth index, consumption, and mother education inequality between poor and non-poor households explains most of the malnutrition gap between the two groups. Results are robust to the different decomposition weighting schemes.

### Robustness of results

It is common and expected to conduct appropriate sensitivity analysis on results obtained to check their robustness either internally or externally. While conducting test of dominance of concentration curve against 45 degree line and Lorenz curve, it is found that in all SES ranking variables and malnutrition indicators, concentration curve dominates 45 degree line and Lorenz curve at the default multiple comparison approach decision rule, 5% significance level, 19 equally spaced quintiles points and rule mca (less strict option). Hence, this study’s results confirm that the concentration curves in all SES ranking variables and malnutrition indicators dominate the 45-degree line and Lorenz curve (lies above). This implies that in all SES and malnutrition indicators, the concentration curve lies above the line of equality, i.e., pro-poor health outcome distribution. However, the results become non dominance of concentration curve over that of 45 degree line and Lorenz curve at the other option, 5% significance level, 19 equally spaced quintile points and rule iup (more strict option). This reflects the fact that the two curves overlap toward the bottom of the SES variable distribution. Further tests on dominance of concentration curve for stunting against wasting, stunting against underweight, and wasting against underweight are conducted. Differences between the cumulative shares of the health and living standards variables at each quintile are also tested.

Although the CI is an appropriate method for measuring inequalities in the health sector, it has implicit in it a particular set of value judgments about aversion to inequality. Accordingly, Wagsta [20] extended concentration index (sensitivity to poverty) is applied. This allows attitudes to inequality to be made explicit, and to see how measured inequality changes as the attitude to inequality changes. It is thus found that inequality rises in all malnutrition indicators when inequality aversion parameters/distributional sensitivity parameter is increased^18^. This assures this study’s results on malnutrition inequalities (with negative sigh of CI) are pro poor^19^ irrespective of the inequality aversion parameters (for details, see Table 13).

**Table 13:** Extended and Symmetric Concentration indices (CI) of malnutrition prevalence by methods

Method	HAZ		WHZ		WAZ	
v, β parameters	1.5	5	1.5	5	1.5	5
Ranking variable -Wealth index						
Extended CI(v)	−.029	−.094	−.019	−.068	−.033	−.112
Symmetric CI(β)	−.038	−.073	−.028	−.043	−.047	−.076
Generalized extended CI(v)	−.327	−.290	−.107	−.106	−.310	−.293
Generalized symmetric CI(β)	−.252	−.486	−.094	−.144	−.262	−.425
Ranking variable -Consumption						
Extended CI(v)	−.026	−.120	−.014	−.022	−.027	−.101
Symmetric CI(β)	−.045	−.060	−.016	−.034	−.040	−.065
Generalized extended CI(v)	−.297	−.370	−.082	−.035	−.256	−.264
Generalized symmetric CI(β)	−.303	−.399	−.054	−.114	−.225	−.366

v = inequality risk aversion parameter, β = degree of sensitivity to extremity or symmetric parameter. If V = 1.5 ➔ more weight to rich, and V = 5➔ more weight to poor, if β =1.5 ➔ more to middle classes, and β = 5➔ more to extreme classes

The normalised concentration indices proposed by Wagsta [40] and Erreygers [41] by specifying the Wagstaff and Erreygers option is also applied while our health variable becomes binary outcome (stunting, wasting and underweight), for details, see Table 5. Results on malnutrition inequalities are still same, i.e. pro-poor. Results using another alternative of attitude to inequality, i.e. symmetric concentration index or `sensitivity to extremity is also tested.

The choice between the symmetric and extended indices is normative. The symmetric index gives equal weight (but with an opposite sign) to individuals that are equally far apart from the pivotal individual with median rank, while the extended index prioritizes the lower regions of the ranking (income) distribution. Erreygers et al. [42] argue that the symmetric index is more concerned about the association between income and health, while the extended concentration index puts priority on the income distribution, and only then analyzes health differences within the prioritized region of the income distribution [35].

To refine results, using decomposition method (as indirect method), results on inequality in malnutrition measured by respected concentration indices for all indicators and SES variables are standardized for age and sex, for details on the results, see see Tables 13 and 14,^20^. Most surveys used for analysis of health sector inequalities in developing countries have complex sample designs. Hence, in this study’s all estimations, appropriate sampling weights to adjust the point estimates for difference in sample size and stratification, and thus for national representative inference is considered. Robust standard errors are also adjusted for each cluster in enumeration areas (primary sampling units).

**Table 14:** Decomposition of child malnutrition inequality-Over all inequality by related groups: Ranking variables -wealth index and consumption

Categories	HAZ-	Score	WHZ-	Score	WAZ-	Score
	Wealth	Consumption	Wealth	Consumption	Wealth	Consumption
All SES inequality	−.045	−.050	−.033	−.016	−.054	−.044
Age-sex standardized CI	−.049	−.047	−.030	−.018	−.050	−.045
Legitimate inequality	.004	−.0009	−.003	.001	−.003	.0007
Illegitimate inequality	−.027	−.034	−.034	−.011	−.030	−.031
Residual	−.022	−.013	.004	−.006	−.021	−.014

under each malnutrition indicators, in the contribution column, the figure in parenthesis represents the percentage contribution

With respect to external validation of this study’s results, it is tried to see some previous studies findings that can be compared. The empirical works on measurement and explanation of socioeconomic inequality in health with longitudinal data conducted by Jones and Lopez [24] supports its findings in dynamics of inequality (not in sign). They demonstrate that over the long-run, represented by a period of 9 years, adverse mental health is more concentrated among the poor. Individual dynamics increase the absolute value of the concentration index of health on income by 10%. Similarly,), on his works of dynamics of socioeconomic -related health inequalities in Australia, Samuel.P, and Calaka Q [43]. shows that socioeconomic related health inequalities have indeed increase over the given period.

There are some evidences that CIs for health outcome are more sensitive to the living standards measure. In due respect, for 19 countries, Wagsta et al. [27] test the sensitivity of the concentration index for child malnutrition to the use of household consumption and a wealth index as the living standards ranking variable. For each of underweight and stunting, the difference between the CIs is significant (10 %) for 6 of 19 comparisons. This suggests that in the majority of countries, child nutritional status is not strongly correlated with inconsistencies in the ranking of households by consumption and wealth. In a similar fashion, Lindelow [44] demonstrates that substantial and significant differences between the CIs for a variety of health services in Mozambique using consumption and an asset index as the living standards measure. In the case of consumption, the concentration index indicates statistically significant inequality in favor of richer households for all services. He also notes that with households ranked by the asset index rather than consumption, the inequality is greater for all services except health center visits, for which the concentration index indicates inequality in utilization in favour of poorer households. Like this study, he argues that the choice of welfare indicator can have a large and significant impact on measured socioeconomic inequalities in a health variable which it depends on the variable examined.

Specifically, a study on Maternal and Child Health Inequalities in Ethiopia conducted by Ambel et al. [12] is a similar work in Ethiopia to this study. Using recent four cross-sectional surveys of DHS implemented in 2000, 2005, 2011, and 2014, they investigate the dynamics of inequalities, employing concentration curves for different years. They find that substantial improvements in health outcomes and health services. Although there still exists a considerable gap between the rich and the poor, the study finds some reductions in inequalities of health services. However, this study’s evidence is different from them, in using longitudinal data and alternative welfare measures, consumption as measure of dynamics of inequality in child undernutrition.

## Conclusion and policy implication

In Ethiopia, undernutrition can best be described in the country as a long-term year round phenomenon due to chronic inadequacies in food combined with high levels of illness in under-five children. Although Ethiopia has already achieved a remarkable progress in reducing under-five mortality in the last decades, undernutrition among children is still a common problem in this country. Thus, socioeconomic inequalities in health outcomes have been of focus in academia and policy spheres for a while now. This study provides new evidence on child undernutrition inequalities in Ethiopia using longitudinal perspective and look at the dynamics of inequality using mobility indices. In all CI computing approaches and SES ranking variables, the CIs are significant with negative value. This implies that in either of short-run or long-run inequality estimates, the burden of unequal distribution of undernutrition remains on the poor. While employing different SES ranking variables, the difference in the CIs is only found significant in case of Height-for-age Z-score (HAZ).

Using standard method, for example, in HAZ, − 0.040 and − 0.070 of CI for wealth index and consumption are scored respectively. It signifies that relatively higher inequality is measured using consumption as ranking variable. This assures the argument of the choice of welfare indicator can have a large and significant impact on measured socioeconomic inequalities in a health variable which it depends on the variable examined. For spatial inequality in malnutrition, CI is also computed for each region and rural-urban. Thus, this study’s results signify that significant difference in inequality of undernutrition is shown across regions while not significant in case of male -female and rural-urban. In this regard, its findings may be helpful in prioritizing resources to reduce inequality and in designing region specific suitable interventions to address such inequity issues. Its inequality results are robust to different measurement scale, inequality aversion parameters/distributional sensitivity parameters, symmetric concentration index or `sensitivity to extremity, and normalization of concentration index. Those results are also standardized for age and sex.

Results on the health-related SES mobility indices computed using Jones and Lopez [24] show that, by the last (third) wave, the short run measure overestimates long run inequality by around 63 and 25%, 65 and 61%, 52 and 41% for HAZ, WHZ and WAZ respectively while wealth index and consumption are considered as ranking variable. Put it differently, this reveals that dynamics decrease the absolute value of the concentration indices of child malnutrition by those given figures. However, results on mobility indices computed based on Allanson et al. [25] approach show that in case of HAZ, the sign of both SES related health mobility index (MH) and health related SES mobility index (MR) are negative in both wealth index and consumption. It implies that individuals face regressive effect (MH < 0) from health change as well as progressive effect from SES ranking change (MR < 0) and the counter balance effect leads to a cumulative effect of no change in inequality change. In other word, persistence of SES inequality in HAZ occurs in the long-run. Similarly, results on WAZ show that MH > 0 and MR < 0. These indicate that individuals face progressive effect in both indices. Thus, it has a cumulative effect of reducing effect on inequality in the long-run which confirms results obtained based on Jones and Lopez [24] approach. While, for WHZ (short -run indicator), there is no clear trend over subsequent waves to put any concluding remarks. Therefore, employing longitudinal perspective rather than weighted average of cross-sectional data is justifiable to see the dynamic of inequality in child malnutrition.

This study’s findings also indicate that there is very limited contribution of the legitimate factor (age) in all malnutrition inequalities which signify that almost all are due to illegitimate factors such as disparity in wealth index, consumption, illness, toilet facility etc. In Height-for-age Z-score (HAZ) and Weight-forage Z score (WAZ), wealth index and mother’s education are the major contributors of socioeconomic related inequality in children undernutrition. While in Weight-for-Height Z-score (WHZ), the loin share is taken by wealth index (30%) and toilet facility (17%). While socioeconomic ranking variable is changed from wealth index to real annual total consumption per adult equivalent, its results indicate that contribution of legitimate factor is a very insignificant which signify that almost all is due to illegitimate factors such as consumption, illness toilet facility etc. In HAZ and WAZ, consumption and mother’s education represent as the major contributors of socioeconomic related inequality in children undernutrition. Though it varies from one undernutrition to other malnutrition indicator, the contribution of regional as well as rural-urban related variation to the inequality is thus smaller by large compare to socioeconomic related variation. Those major contributors to the inequality (mother’s education level, wealth index and consumption expenditure) are also found statistically significant (with expected sign).

Results on Oaxaca decomposition shows that the explained and unexplained (coefficient) effects are only significant in case of HAZ and WAZ while interaction effects are insignificant in all indicators. Other results also show that the explained (covariate) effect is dominant while the coefficients effects are relatively low in the all malnutrition indicators. SES variables such as wealth index, consumption, and mother education inequality between poor and non-poor households explains most of the malnutrition gap between the two groups. These imply that in both SES ranking variables, the bulk of inequality in malnutrition is caused by inequality in SES in which it disfavour the poor in both cases. This calls for enhancing the policy measures that narrow socioeconomic gaps between groups in the population and targeting on early childhood intervention and nutrition sensitive. These findings thus helps to strengthen the different nutrition improving sensitive and social protection programs such as Productive Safety Net Program (PSNP) in the country which are designed to narrow the socioeconomic related inequality and reduce child malnutrition prevailed across the country.

## Data Availability

Data for the study comes from the Ethiopia Socioeconomic Survey (ESS) collected jointly by the Central Statistical Agency (CSA) of Ethiopia and the World Bank as part of the Living Standard Measurement Study-Integrated Surveys on Agriculture (LSMS-ISA). It is a longitudinal survey with three waves (2011/12, 2013/14 and 2015/16).

## Appendix

**Table 15:** Three fold decomposition of mean difference of child undernutrition between poor (bottom 60%) and non-poor (top 40%)

Variables /undernutrition levels	HAZ	WHZ	WAZ
Overall differential			
Mean prediction for Rich (R)	−1.21*** (.060)	−.429*** (.051)	−1.10*** (.050)
Mean prediction for Poor (P)	−1.52*** (.056)	−.530*** (.042)	−1.35*** (.041)
Row Difference(R-P)	.305*** (.073)	.100* (.059)	.249*** (.056)
due to Endowments (explained) -E	.265* (.146)	.039 (.118)	.313*** (.097)
due to Coefficients (unexplained) -C	.188* (.105)	.025 (.079)	.097 (.079)
due to Interactions (CE)	−.147 (.167)	.036 (.130)	−.162 (.113)
Observations(N)	8686	8132	9426

Significance level: ***, ** and * is at 1%, 5 and 10% respectively and Std. Errors(in parenthesis) are adjusted for each clusters in ea_id (enumeration areas or primary sampling units)

**Table 16:** Summary of decomposition results: Decomposition results of the poor-non-poor gap in malnutrition with different weighting schemes

D	0	1	0.5	.276	*
HAZ					
Unexplained	0.040	0.188	0.114	0.081	0.066
Explained	0.265	0.117	0.191	0.224	0.239
% unexplained	13.2	61.6	37.4	26.6	21.7
% explained	86.8	38.4	62.6	73.4	78.3
WHZ					
Unexplained:	0.061	0.025	0.043	0.051	0.027
Explained	0.039	0.075	0.057	0.049	0.074
% unexplained	61.0	25.1	43.1	51.1	26.7
% explained	39.0	74.9	56.9	48.9	73.3
WAZ					
Unexplained	−0.065	0.098	0.016	−0.020	0.026
Explained	0.314	0.152	0.233	0.269	0.223
% unexplained	−26.0	39.2	6.6	−8.0	10.3
% explained	126.0	60.8	93.4	108.0	89.7

D in 4th column = relative frequency of high group, * reference: pooled model over both categories

**Table 17:** Prevalence of stunting, wasting and underweight by region and rural/urban

Groups	Stunting	Wasting	Underweight
Rural -urban			
Rural	3214 (30.90)	1007(9.68)	2479(23.83)
Small town	183(22.56)	58 (7.15)	122(15.04)
Regions			
Tigray	351(35)	131(13.06)	326 (32.50)
Amhara	624(33.91)	171 (9.29)	449 (24.40)
Oromia	637(26.91)	181 (7.65)	458 (19.35)
SNNP	1050(33.35)	231(7.34)	707(22.46)
Other Regions	735(25.75)	351(12.30)	661(23.16)

All values in parentheses are in percentage. Others includes samples from Afar, Somalie, Gambelia, Benshangul Gumuz, Harari and Diredwa which are all together nationally representative

**Table 18:** Decomposition of child malnutrition inequality (CI) based on Fixed effect estimator: Ranking variable Wealth index

Regressors (k)	HAZ					WHZ				WAZ		
	*β*_*k*_	Elasticity	CI	Contribution	*β*_*k*_	Elasticity	CI	Contribution	*β*_*k*_	Elasticity	CI	Contribution
Age	.006* (.003)	.17	−.02	−.01(.11)	−.015***(.003)	−.85	−.03	. 03(−.76)	.006*(.003)	.21	−.03	−.01(.11)
Age-square	−.000**(.000)	−.11	−.05	.01(−.12)	−.000*(.000)	.51	−.05	−.03(.83)	.000(.000)	.01	−.05	−.00(.01)
Sex	.433***(.114)	.13	.02	.00(−.06)	.131*(.076)	.08	.02	.00(−.05)	.327***(.081)	.12	.02	.00(−.04)
Illness incidence	.142**(.055)	.01	.05	.00(−.02)	.122***(.043)	.02	.06	.00(−.04)	.160***(.038)	.02	.05	.00(−.02)
Water availability	−.019(.067)	−.00	.19	−.00(.01)	.055(.049)	.02	.19	.00(−.09)	.025(.054)	.00	.19	.00(−.01)
Toilet type	.012(.023)	.05	−.02	−.00(.02)	.004(.018)	.03	−.02	−.00(.02)	.037**(.015)	.16	−.02	−.00(.07)
Health post	−.144* (.084)	−.09	.00	.00(−.00)	.006 (.076)	.01	.00	2.5(−.00)	−.059(.070)	−.05	−.00	.00(−.00)
Mother educ	−.033(.058)	−.01	.32	−.00(.06)	−.033(.034)	−.02	.31	−.01(.16)	.004(.040)	.00	.32	.00(−.01)
Household size	−.042(.027)	−.16	.01	−.00(.03)	.031(.023)	.23	.01	.00(−.04)	.001(.018)	.01	.01	.00(−.00)
Household sizeU5	−.037(.038)	−.03	−.01	.00(−.01)	−.023(.032)	−.04	−.00	.00(−.00)	−.096***(.026)	−.10	−.00	.00(−.00)
Wealth index	0.114***(.033)			.05(−1.12)	−.028(.022)			−.02(.75)	.062***(.021)			03(−.58)
Quntile 1		−.04	−.78	.04(−.80)		.02	−.80	−.01(.53)		−.03	−.80	.02(−.43)
Quntile 2		−.04	−.40	.02(−.38)		.01	−.40	−.00(.07)		.03	−.40	.01(−.23)
Quntile 3		−.02	.02	−.00(.01)		.01	.01	.00(−.00)		−.02	.01	−.00(.00)
Quntile 4		−01	.41	−.00(.06)		−.01	.41	−.00(.15)		−.01	.42	−.00(.08)
Residual				−.094(2.07)				−.007(.22)				08(1.7)
Observation	8686				8132				9426			
R^2^	0.025				0.025				0.040			

Under each malnutrition indicators, in the contribution column, the figure in parenthesis represents the percentage contribution. Each figure is rounded to two digits only. Hence, point zero zero doesn’t mean that it is actually zero, it is rather rounded value

**Table 19:** Decomposition of child malnutrition inequality (CI) based on Fixed effect estimator: Ranking variable -Real annual total consumption per adult equivalent

Regressors (k)	HAZ					WHZ				WAZ		
	β_*k*_	Elasticity	CI	Contribution	β_*k*_	Elasticity	CI	Contribution	β_*k*_	Elasticity	CI	Contribution
Age	.003(.003)	.08	.01	.00(−.01)	−.014(.003)	−.81	−.00	.00(−.15)	.005*(.002)	.17	.01	.00(−.02)
Age-square	−.000*(.000)	−.10	.01	−.00(.02)	−.000**(.000)	.53	−.00	−.00(.08)	.000(.000)	.00	.01	.00(−.00)
Sex	.453***(.115)	.14	−.00	−.00(.00)	.119(.080)	.07	.00	.00(−.01)	.332***(.079)	.12	.00	3.7(−.00)
Illness incidence	.149***(.055)	.01	−.00	−.00(.00)	.129***(.043)	.02	.00	.00(−.00)	.163***(.038)	.02	−.00	−.01(−.00)
Water availability	−.029(.069)	−.00	.05	−.00(.00)	.052(.048)	.01	.04	.00(−.04)	.027(.057)	.00	.04	.00(−.00)
Toilet type	.004(.023)	.01	−.00	−.00(.00)	.010(.017)	.07	−.00	−.00(.01)	.036**(.016)	.16	−.00	−.00(.00)
Health post	−.163* (.090)	−.10	.01	−.00(.01)	.009(.080)	.01	.01	.00(−.00)	−.079(.075)	−.06	.01	−.00(.00)
Mother educ	−.015(.056)	−.00	.26	−.00(.02)	−.032(.034)	−.02	.26	−.00(.24)	−.000(.039)	−.00	.26	−5.2(.00)
Household size	−.047* (.028)	−.18	−.03	.01(−.12)	0.031(.024)	.23	−.03	−.01(.47)	.007(.019)	.03	−.03	−.00(.02)
Household sizeU5	−.028 (.040)	.01	−.01	.00(−.00)	−.020(0.34)	−.03	−.01	.00(−.01)	−.088***(.026)	−.09	−.01	.00(−.02)
Consumption	−.103**(.044)			−.02(.39)	.004(.029)			00(−.15)	−.028(.031)			−.01(.11)
Quntile 1		.02	−.80	−.01(.30)		−.01	−.80	.01(−.31)		.01	−.80	−.00(.10)
Quntile 2		.01	−.40	−.00(.09)		−.00	−.40	.00(−.06)		−.00	−.40	.00(−.01)
Quntile 3		.01	.00	.00(−.00)		.02	.01	.00(−.01)		.01	.01	.00(−.00)
Quntile 4		.00	.40	.00(−.01)		−.01	.41	−.008(.23)		−.00	.41	−.00(.02)
Residual				−.033(.69)				−.01(.56)				−.040(.89)
Observation	8505				7973				9229			
R^2^	0.018				0.027				0.037			

under each malnutrition indicators, in the contribution column, the figure in parenthesis represents the percentage contribution. Each figure is rounded to two digits only. Hence, point zero zero doesn’t mean that it is actually zero, it is rather rounded value

## Footnotes

1 Socioeconomic determinants for health outcome are either interrelated or longitudinal in nature.

2 Chronic inequalities might call for policies to tackle the structural problems that trap some individuals in deprivation and ill-health while transitory episodes might demand measures such as improvements in access to and delivery of acute health services or temporary welfare assistance. Thus, further work towards a comprehensive framework for modeling and evaluating the impact of specific policies and interventions on health inequalities is required to provide a consistent basis for resource allocation and welfare policies.

3 Little attention has focused on measuring health mobility or whether the health of the poor is improving relative to the rich over time. This is an important issue since significant income-related inequalities in health have persisted, and even increased, in countries over the last decade in spite of considerable improvements in average health status [19]. However, measures that do not exploit the advantages of real longitudinal data (i.e., that do not follow individuals over time) are unable to distinguish transitory inequalities (short episodes of ill-health and poverty) from ongoing structural socioeconomic and health-related deprivation. In particular, dynamic measures allow one to distinguish between transitory and chronic health inequalities and to characterize processes of inequality change.

4 The justification behind this is that in developing countries, formal employment is less common. Many households have multiple and continually changing sources of income, and home production is more widespread. In these contexts, it is generally far easier to measure consumption than income.

5 ESS began as ERSS (Ethiopia Rural Socioeconomic Survey) in 2011/12. The first wave of data collection in 2011/12 included only rural and small town areas. The survey name dropped the word rural in the second wave of data collection when the sample was expanded to include all urban areas. The urban supplement was done in such a way to ensure that the ESS data can provide nationally representative estimates.

6 Categorized based on WHO standard given for toilet type. It includes Flush toilet -private, Flush toilet-shared, Pit latreen- private ventilated, Pit lantreen-shared ventilated, Pit lantreen-private -ventilated, Pit lantreen-shared not.

7 Included 35 asset items such as Kerosene stove, Butane Gas Stove, Electric Stove, Blanket/Gabi, Mattress and /or Bed, Wrist watch/clock, Fixed line telephone, Mobile telephone, Radio/ radio and tape/ tape, Television, CD/ VCD/ DVD / Video Deck, Satellite Dish, Sofa set, Bicycle, Motorcycle, Cart (hand pushed), Cart (animal drawn), Sewing machine, Weaving equipment, Mitad-Electric, Mitad-power saving (modern), Refridgerator, Private car, Jewels (Gold and silver), Wardrobe, Shelf for storing goods, Biogas stove (pit), Water storage pit, Mofer and Kember, Sickle (Machid), Axe (Gejera), Pick Axe (Geso), Plough (traditional), Plough (modern) and Water Pump

8 In all surveys, consumption and expenditure information was collected on a limited number of items. The consumption and expenditure information was collected within the household questionnaire during the third visit to the household in both surveys; this occurred between January and March 2012 for ESS1 and between February and April 2014 for ESS2. Information was collected for 25 food items consumed over the last 7 days2, 11 basic household goods (matches, batteries soap, etc.) over the past month, and 12 other expenditures (men’s clothing, linens, etc.) over the past 12 months.

9 Bases on [33]1 proposed equivalences on nutritional (caloric) requirements of different ages for both men and women

10 With repeated cross-section or panel data, one can use the command to compare inequality across periods. The command can also be used to estimate rank-dependent indices of univariate inequality, such as the Gini and generalized Gini.

11 There is complete immobility when the relative incomes of all individuals remain constant over time. However, as income profiles deviate further from this extreme, income mobility increases. If incomes are not completely immobile, inequality tends to decline as the length of the measurement period increases (Shorrocks’, 197).

12 With respect to interpretation of decomposition results, one should carefully realize that though decomposition methods are based on regression analyses, there are two possible cases: First, if regressions are purely descriptive, they reveal the associations that characterize the health inequality. Then inequality is explained in a statistical sense but implications for policies to reduce inequality are limited. Second, if data allow identification of causal effects, the factors that generate the inequality are identified. Then, it is possible to draw conclusions about how policies would impact on inequality. Hence, estimation technique and model that t for our purpose is selected with this context.

13 Depending on our estimator’s choice, α_*i*_ can be random or non-random if it is random effect or fixed effect estimator respectively

14 The residual component captured by the last term reflects the income-related inequality in health that is not explained by systematic variation in the regressors such as by income, which should approach zero for a well-specified model.

15 This study also computed concentration index while the health variable is binary outcome (stunted, wasted, and underweighted. The results are almost similar

16 Alternatively, using binary outcomes as dependent variables (stunted, wasted and under-weighted option), we also estimate our regression model by OLS and pooled probit and results are more or less similar.

17 Though specific results based on those alternative estimators are different from that of random effect, the contribution of socioeconomic factors to the observed inequalities in malnutrition is still dominant.

18 As inequality aversion parameters/distributional sensitivity parameter, the more weight is attached to health of poor individuals in the distribution and the weight attached to the health of people who are above the 55th percentile decreases.

19 In terms of sign concentration index, results using standard concentration index with regular parameters are same as while we alter inequality aversion parameters above regular parameters. In both option, inequalities in malnutrition are pro-poor.

20 As such, by incorporating various confounding variables, all computed concentration indices are standardized using indirect methods (see O’Donnell et al. [34] Chapter 8 for details)

## References

[1] Black RE, Victora CG, Walker SP (2013). Maternal and child undernutrition and over-weight in low-income and middle income countries. Lancet..

[2] Abuya BA, Ciera JM, Kimani-M E (2012). Effect of mother's education on child's nutritional status in the slums of Nairobi. BMC Pediatr..

[3] Glewwe P, Miguel EA (2007). The impact of child Health and nutrition on education in less developed countries. Hand book of development economics.

[4] United Nations Children’s Fund, UNICEF (2017). Child malnutrition estimates.

[5] Central Statistical Authority (Ethiopia), CSA and ORC Macro (2011). Ethiopia Demographic and Health Survey 2011.

[6] Wagsta A, Caryn B, Leander RB (2014). Progress toward the Health MDGs: are the poor being left behind?. Word Bank policy research working paper 6894.

[7] Barros FC, Cesar GV, Robert S, Davidson G (2010). Socioeconomic inequities in the health and nutrition of children in low/middle income countries. Rev Saúde Pública..

[8] McKinnon B, Harper S, Kaufman JS, Bergevin Y (2014). Socioeconomic inequality in neonatal mortality in countries of low and middle income: a multi-country analysis. Lancet Glob Health..

[9] Quentin W, Abosede O, Aka J, Akweongo P, Dinard K, Ezeh A (2014). Inequalities in child mortality in ten major African cities. BMC Med..

[10] UNICEF (2015). Updated National Equity Situation Analysis on Chirdren and Women in Ethiopia.

[11] Alemu ZA, Ahmed AA, Yalew AW, Birhanu BS (2016). Nonrandom distribution of child under-nutrition in Ethiopia: spatial analysis from the 2011 Ethiopia demographic and health survey. Int J Equity Health..

[12] Ambel A, Andrews C, Bakilana A, Foster E, Khan Q, Wang H (2015). Maternal and child Health inequalities in Ethiopia. Policy research working paper 7508.

[13] Asfaw M, Wondaferash M, Taha M, Dube L (2015). Prevalence of under-nutrition and associated factors among children aged between six to fty nine months in Bule Hora district, South Ethiopia. BMC Public Health.

[14] Derek H (2014). An analysis of trends and determinants of child under-nutrition in Ethiopia, 2010–2011, ESS, IFPRI working paper 70.

[15] Hailie D, Azage M, Mola T, Rainey R (2016). Exploring spatial variations and factors associated with childhood stunting in Ethiopia: spatial and multilevel analysis. BMC Paediatrics..

[16] Misgan L, Taye A, Yohannes H (2016). Determinants of Child Malnutrition among Agro Pastorals in Northeastern Ethiopia: A Cross-Sectional Study. Health Sci J.

[17] Zewdie T, Abebaw D (2013). Determinants of Child Malnutrition: Empirical Evidence from Kombolcha District of Eastern Hararghe Zone, Ethiopia. Quarter J Int Agric.

[18] WB., World Bank (2012). Health equity and financial protection data sheet - Ethiopia.

[19] van Doorsalaer E, Koolman X (2004). Explaining the differences in income-related health inequalities across European countries. Health Econ.

[20] Wagsta A (2002). Theme papers poverty and health sector inequalities. Bull World Health Organ..

[21] Thomson L, Jess D, Andre R, Michael T (2014). Addressing child un-dernutrition: evidence review.

[22] Wagsta A, Pact P, Doorslae E (1991). On The Measurement Of Inequalities In Health. Sm Sci Med..

[23] Kakwani N, Wagsta A, Van Doorslaer E (1997). Socioeconomic inequalities in health: measurement, computation and statistical inference. J Econ..

[24] Jones AM, Lopez A (2004). Measurement and explanation of socioeconomic inequality in health with longitudinal data. Health Econ..

[25] Allanson P, Ulf-G G, Dennis P (2010). Longitudinal analysis of income-related health inequality. Dundee Discussion Working Paper No. 214, 2008 ISSN:1473-236X.

[26] DeOnis EA (2000). Frongillo, Monika B. Is malnutrition declining? An analysis of changes in levels of child malnutrition since 1980 Mercedes de. Bull World Health Organ..

[27] Wagsta A, van Doorslaer E, Watanabe N (2003). On decomposing the causes of health sector inequalities with an application to malnutrition inequalities in Vietnam. J Econ..

[28] Fotso J (2006). Child health inequities in developing countries: differences across urban and rural areas. Int J Equity Health..

[29] Bredenkamp LR, Ellen V (2014). Persistent inequalities in child undernutrition: evidence from 80 countries, from 1990 to today. Int J Epidemiol..

[30] Skaftun EK, Ali M, Norheim (2014). Understanding Inequalities in Child Health in Ethiopia: Health Achievements are Improving in the Period 20002011. PLoS One.

[31] World Health organization (1995). The world Health Report 1995: Bridging the gap.

[32] World Health Organization, WHO (2006). WHO child growth standards: length/height-for-age, weight-for-age, weight-for-length, weight-for-height and body mass index-for-age: methods and development.

[33] Dercon S, Pramila K. Changes in povery in rural Ethiopia 1989-1995: Measurement, robustness tests and decomposition. CES-Discussion paper series (DPS). 1998;98:19.

[34] O'Donnell O, van Doorslaer E, Wagsta A, Lindelow M (2008). Analyzing Health equity using household survey data: a guide to techniques and their implementation.

[35] O'Donnell O, O'Neill S, Van Ourti T, Walsh B (2016). Conindex: estimation of concentration indices. Stata J..

[36] Shorrocks A (1978). Income inequality and income mobility. J Econ Theory..

[37] Lavy V, Strauss J, Thomas D (1996). de Vreyer, Philippe. Quality of health care, survival and health outcomes in Ghana. J Health Econ..

[38] Thomas D, Lavy V, Strauss J (1996). Public policy and anthropometrics outcomes in the Côte D'Ivoire. J Public Econ..

[39] Oaxaca R (1973). Male-female wage differentials in urban labor markets. Int Econ Rev..

[40] Wagsta A (2005). The bounds of the concentration index when the variable of interest is binary, with an application to immunization inequality. Health Econ..

[41] Erreygers G (2009). Can a single indicator measure both attainment and shortfall inequality?. J Health Econ..

[42] Erreygers G, Clarke P, Van Ourti T (2012). Mirror, mirror, on the wall, who in this land is fairest of all? Distributional sensitivity in the measurement of socioeconomic inequality of health. J Health Econ.

[43] Samuel P, Calaka Q (2015). Dynamics of socioeconomic -related health inequalities in Australia. A master thesis: Lund University.

[44] Lindelow M (2006). Sometimes more equal than others: how Health inequalities depend upon the choice of welfare Indicator. Health Econ..

